# Isoform-specific functions of Mud/NuMA mediate binucleation of *Drosophila* male accessory gland cells

**DOI:** 10.1186/s12861-014-0046-5

**Published:** 2014-12-20

**Authors:** Kiichiro Taniguchi, Akihiko Kokuryo, Takao Imano, Ryunosuke Minami, Hideki Nakagoshi, Takashi Adachi-Yamada

**Affiliations:** Department of Life Science, Faculty of Science, Gakushuin University, Tokyo, 171-8588 Japan; Institute for Biomolecular Science, Gakushuin University, Tokyo, 171-8588 Japan; Department of Biology, Graduate School of Science, Kobe University, Kobe, 657-8501 Japan; Graduate School of Natural Science and Technology, Okayama University, Okayama, 700-8530 Japan

**Keywords:** *Drosophila*, Male accessory gland, Binucleation, Cytokinesis, Central spindle, Cell rounding, Spindle orientation, Mud

## Abstract

**Background:**

In standard cell division, the cells undergo karyokinesis and then cytokinesis. Some cells, however, such as cardiomyocytes and hepatocytes, can produce binucleate cells by going through mitosis without cytokinesis. This cytokinesis skipping is thought to be due to the inhibition of cytokinesis machinery such as the central spindle or the contractile ring, but the mechanisms regulating it are unclear. We investigated them by characterizing the binucleation event during development of the *Drosophila* male accessory gland, in which all cells are binucleate.

**Results:**

The accessory gland cells arrested the cell cycle at 50 hours after puparium formation (APF) and in the middle of the pupal stage stopped proliferating for 5 hours. They then restarted the cell cycle and at 55 hours APF entered the M-phase synchronously. At this stage, accessory gland cells binucleated by mitosis without cytokinesis. Binucleating cells displayed the standard karyokinesis progression but also showed unusual features such as a non-round shape, spindle orientation along the apico-basal axis, and poor assembly of the central spindle. Mud, a *Drosophila* homolog of NuMA, regulated the processes responsible for these three features, the classical isoform Mud^PBD^ and the two newly characterized isoforms Mud^L^ and Mud^S^ regulated them differently: Mud^L^ repressed cell rounding, Mud^PBD^ and Mud^S^ oriented the spindle along the apico-basal axis, and Mud^S^ and Mud^L^ repressed central spindle assembly. Importantly, overexpression of Mud^S^ induced binucleation even in standard proliferating cells such as those in imaginal discs.

**Conclusions:**

We characterized the binucleation in the *Drosophila* male accessory gland and examined mechanisms that regulated unusual morphologies of binucleating cells. We demonstrated that Mud, a microtubule binding protein regulating spindle orientation, was involved in this binucleation. We suggest that atypical functions exerted by three structurally different isoforms of Mud regulate cell rounding, spindle orientation and central spindle assembly in binucleation. We also propose that Mud^S^ is a key regulator triggering cytokinesis skipping in binucleation processes.

**Electronic supplementary material:**

The online version of this article (doi:10.1186/s12861-014-0046-5) contains supplementary material, which is available to authorized users.

## Background

Most eukaryotic cells contain only a single nucleus because the karyokinesis in the M phase of the cell cycle is followed by cytokinesis. In certain cells, however, such as cardiomyocytes and hepatocytes, cytokinesis does not always occur, which results in cells containing two nuclei [1,2]. Sarcomere assembly is a possible factor repressing cytokinesis in cardiomyocytes [1,3], and insulin signaling plays a part in the generation of binucleate hepatocytes [2].

The production of binucleate cells is thought to result from the repression of certain phases of cytokinesis, such as formation of the contractile ring and ingression of the cleavage furrow. Cytokinesis occurs only when there is sufficient activation of Rho GTPase at the division plane. After chromosome segregation, a prominent bundle of microtubules, called the central spindle, forms between the spindle poles [4-6]. The centralspindlin complex, consisting of kinesin-6 and RhoGAP, moves toward the plus ends of the microtubules, corresponding to the cell equator, and associates with RhoGEF [7]. The RhoGEF thus specifically activates Rho GTPase at the division plane. Rho signaling activates effector proteins, such as diaphanous and Rho kinase, that in turn activate the formation of the actin contractile ring that completes cell division by pinching the daughter cells apart [8]. Loss of this cytokinesis machinery results in incomplete cytokinesis and produces binucleate cells [7,9,10]. There is, however, no solid evidence that normal binucleation events are regulated by inhibiting the functions of cytokinesis components, and little is known about the key regulators repressing the formation of cytokinesis machinery during binucleation. On the other hand, recent studies have shown a link between binucleation and inhibition of the cytokinesis machinery in cancer cells [11,12].

**Figure 1:**
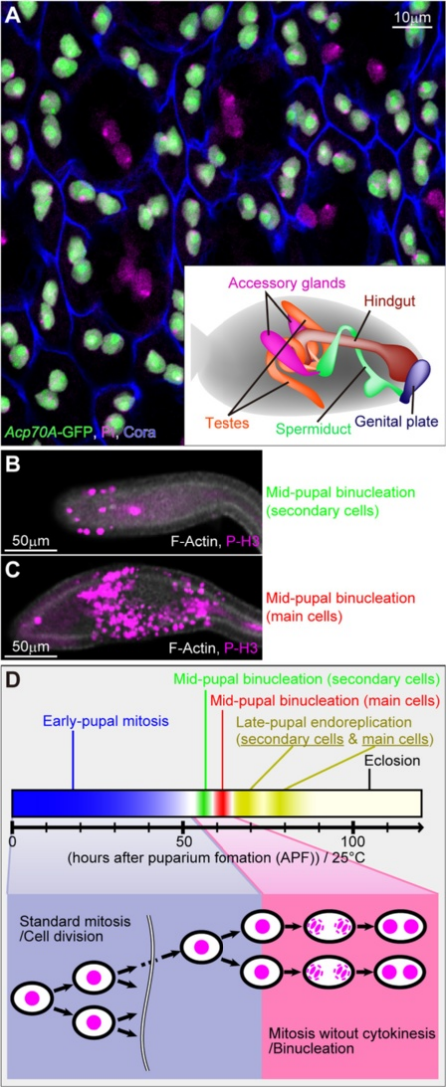
**Synchronous binucleation of** ***Drosophila*** **male accessory gland cells occurs in the pupal stage. (A)** Adult accessory gland epithelium labeled as indicated at the bottom left. Main cells (nuclei stained both green and magenta) and secondary cells (nuclei stained only magenta) are shown. The inset at the bottom right depicts an adult male abdomen (gray) and the reproductive systems around the hindgut. Posterior is to the right. Scale bar, 10 μm. **(B and C)** Synchronous entry into M phase in secondary **(B)** and main **(C)** cells in the accessory glands during mid-pupal binucleation stages. Labels as indicated at the bottom right. Scale bars, 50 μm. **(D)** Schematic diagram showing cell cycle transition of epithelial cells in accessory gland during the pupal stage.

To investigate the mechanism by which cytokinesis is skipped during binucleation, we used as a model system the *Drosophila* male accessory gland, which produces seminal fluid proteins promoting reproductive success, such as the sex peptide Acp70A [13,14]. The exocrine epithelial cells in the male accessory gland, both the main cells and the secondary cells, are obviously binucleate (Figure 1A) [15]. We previously showed that binucleation increases the plasticity of the cell shape, thereby enabling the volume of the accessory gland cavity to change [16], but the mechanisms of binucleation have remained unclear.

In the work reported here, we investigated the binucleation event in the accessory gland primordia, which was characterized by synchronous entry into the M phase after a cell-cycle-arrested interval during the mid-pupal developmental stage following standard cell proliferation in the early stage. We found that the binucleation results not from cell fusion but from mitosis without cytokinesis. We examined the mechanisms of binucleation by focusing on various morphological features different from those of standard dividing cells. We propose that isoform-specific functions of the microtubule binding protein Mud, a *Drosophila* homolog of NuMA, are the key regulators in binucleation of the *Drosophila* male accessory gland cells.

## Results

### Accessory gland epithelial cells are binucleated synchronously in the mid-pupal stage by mitosis without cytokinesis

**Figure 2:**
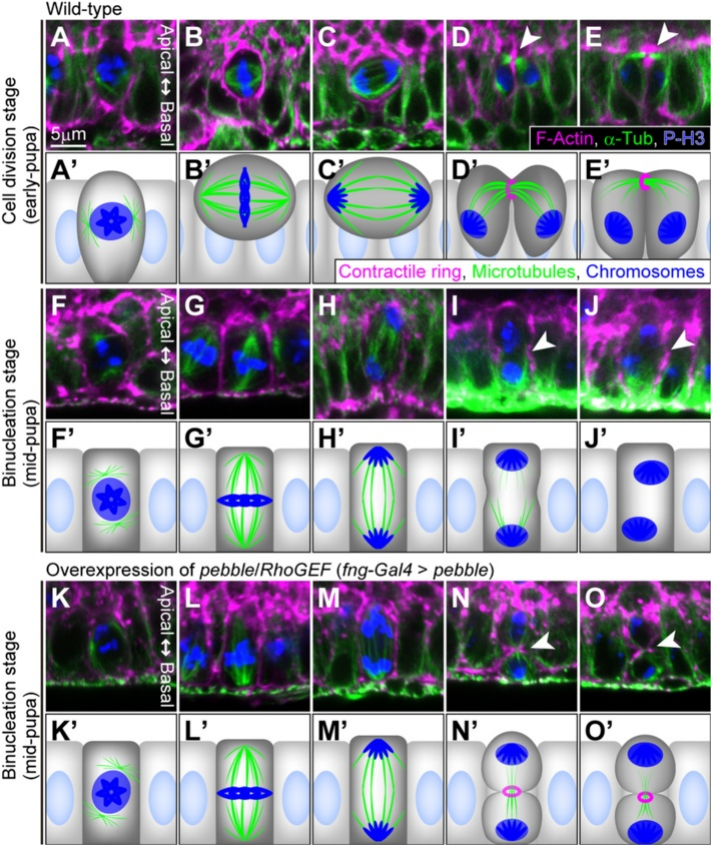
**Central spindle and contractile ring are not formed during binucleation.** Photomicrographs showing cross-sectional views of cells **(A–O)** and their schematic diagrams **(A’–O’)** are arrayed from left to right according to the M phase progression. **(A–E)** Main cells during cell division stage in early pupa (30–35 hours APF). **(F–J)** Main cells during binucleation stage in mid-pupa (60–65 hours APF). **(K–O)** Binucleation-stage main cells in which *pebble* was overexpressed just before binucleation. Arrowheads in **(D, E, I, J, N and O)** indicate equatorial planes in late anaphase and telophase during cell division and binucleation. Cells are labeled as indicated at the bottom of **(E)**. Scale bar in **(A)**, 5 μm, is applicable to **(A–O)**.

We first determined whether binucleation of the accessory gland epithelial cells is a result of skipping cytokinesis (as in cardiomyocytes). We observed the developmental stages and M-phase entry by using an antibody against phospho-histone H3 (P-H3), a marker for M-phase chromatin. Until 50 hours after puparium formation (APF), the accessory gland epithelial cells randomly entered the M phase but did not produce binucleate cells (Additional file 1: Figure S1A–E, A’–E’ and J) (Figure 1D). That is, standard cell division occurred. Subsequently, the cells arrested their cell cycle and delayed their M-phase entry for about 5 hours (50-55APF) (Additional file 1: Figure S1F and F’) (Figure 1D). The secondary cells then entered the M phase at 55 hours APF (Figure 1B and D) (Additional file 1: Figure S1G and G’), and the main cells entered the M phase at 60 hours APF (Figure 1C and D) (Additional file 1: Figure S1H and H’). We also found that the mitotic wave for binucleation in the main cell population initiated at the middle zone of the accessory gland lobe and propagated to the proximal and distal parts (Additional file 1: Figure S2). These results indicate a unique cell cycle regulation in this organ development. Importantly, the synchronous entries into the M phase accompanied the production of binucleate cells (Additional file 1: Figure S1K and Figure S2). No cytokinesis was evident in this M phase (Figure 2F–J and F’–J’). After binucleation, the accessory gland epithelial cells did not enter a subsequent M phase (Additional file 1: Figure S1I and I’, Figure S3) but showed a single round of the S phase, indicated by PCNA-GFP labeling (Additional file 1: Figure S3), indicating that endoreplication occurred (Figure 1D). Thus the accessory gland epithelial cells, both secondary and main cells, became octaploid cells with two tetraploid nuclei. In the following section, we describe our examination of binucleation in the main cells. The secondary cells probably binucleated in the same way the main cells did.

### Central spindle assembly and actin-contractile ring formation are inhibited during binucleation

**Figure 3:**
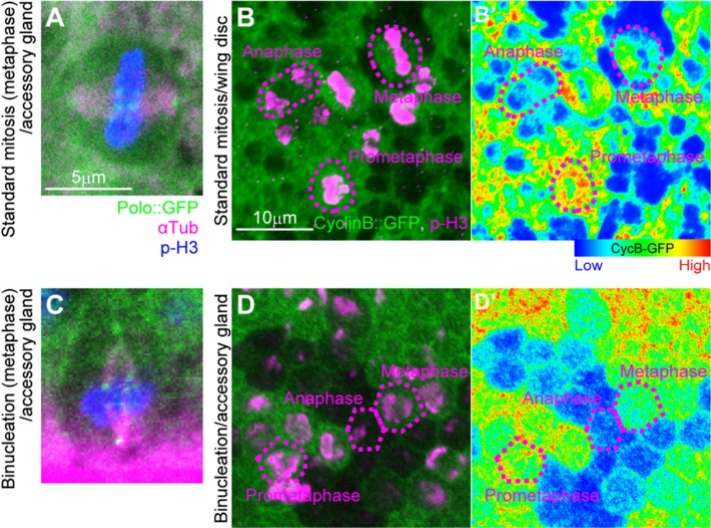
**Metaphase spindle formation and metaphase-anaphase transition are normal during binucleation. (A and C)** Localization of polo to kinetochores in main cells in metaphase during cell division in early pupa **(A)** and during binucleation in mid-pupa **(C)**. Cells are labeled as indicated at the bottom of **(A)**. *En face* views **(A)** and cross-sectional views **(C)**. Scale bar, 5 μm, is applicable to **(A and C)**. **(B, B’, D and D’)** Levels of cyclin B. *En face* images of the wing disc epithelium in third-instar larva as an example of proliferating tissue **(B and B’)** and the accessory gland epithelium in mid-pupa as an example of binucleating tissue **(D and D’)**. Cells in **(B and D)** are labeled as indicated at the bottom right of **(B)**. Intensities of cyclin B::GFP in **(B)** and **(D)** are represented by a rainbow-color scale, with red meaning high intensity and blue meaning low intensity. Magenta dashed lines indicate outlines of mitotic cells in various M-phase subphases. Scale bar in **(B)**, 10 μm, is applicable to **(B, B’, D and D’)**.

We next identified the cytological differences between standard cell division and binucleation in order to obtain clues to the mechanisms of cytokinesis skipping. For this purpose, we compared cell division in the early pupal accessory gland primordium and binucleation in the mid-pupal one (Figure 1D). We focused on the following three differences. First, during the standard cell division in the early stage of accessory gland development, the M-phase cells were apically extruded and rounded (Figure 2A–C and A’–C’), as is widely found in the standard epithelia [17]. During binucleation, however, cells were retained in the epithelial monolayer and did not show rounding (Figure 2F–H and F’–H’ compared with A–C and A’–C’). Second, the spindle orientation in standard mitosis was parallel to the epithelial plate (Figure 2A–C and A’–C’) [17]. The spindle formed during binucleation, in contrast, was always oriented perpendicular to the epithelial plate (Figure 2F–H and F’–H’). Third, in standard cell division, the central spindles between segregated chromatids arose from anaphase to telophase, and then an actin contractile ring formed at the division plane (Figure 2D,E,D’ and E’) [4-6,8]. During binucleation, in contrast, the central spindle was not properly assembled (Figure 2I,J,I’ and J’). Consequently, the subsequent formation of the contractile ring was also incomplete at telophase (Figure 2J and J’), although the cleavage furrow was slightly formed at late anaphase (Figure 2I and I’). On the other hand, other components of the mitotic spindle of binucleating cells looked normal, including the metaphase spindle (Figure 2G and G’ compared with B and B’) [6] and kinetochore microtubules (Figure 3C compared with A) [6,18]. Also apparently normal in the binucleating cells was the M phase progression indicated by the relationship between chromatid segregation (Figure 2G,H,G’ and H’ compared with B,C,B’ and C’) [18] and the decay of cyclin B (Figure 3D and D’ compared with B and B’) [19]).

### Forced activation of Rho GTPase in binucleation stage produces actin contractile ring

Looking at the above three differences for clues to the mechanism of binucleation in the experiments intended to produce artificially rounded cells, we saw that cell extrusion and rounding did not seem to affect binucleation. In the binucleation stage neither overexpression of Sterile20-like kinase, which is related to cortical rigidity and cell rounding in standard cell division [20,21], nor knockdown of the adherens junction protein *D*E-cadherin encoded by *shotgun* [22], which maintains epithelial stability, led to cytokinesis (Additional file 1: Figure S4A–D) (Table 1). Binucleation also seemed to not be affected by the orientation of the mitotic spindle. This is because disruption of vertical spindle orientation in the binucleation stage by using a mutant for the centrosome protein Centrosomin or Sas-4 did not cause ectopic cytokinesis progression (Additional file 1: Figure S4E–H) (Table 1) [23-25]. On the other hand, in *centrosomin* and *Sas-4* mutants, weak assembly of the central spindle and an abnormal contractile ring could be observed at low frequencies (6% in *centrosomin*^*HK21*^ and 9% in *Sas-4*^*S2214*^). However, we found that these induction-of-cytokinesis features were observed even in the cells showing normal (vertical) spindle orientation. Thus these results mean that contribution of the centrosome to the cytokinesis skipping during binucleation may be nonessential or even trivial.

**Table 1:** **Effect of various genetic manipulations on central spindle assembly and contractile ring formation during binucleation**

**Manipulation**	**Genotype**	**Frequencies of phenotypes in mitotic behavior**	
		**Central spindle assembly**	**Contractile ring formation**
None (wild-type)	*Canton-S*	0% (N = 42, 7)	0% (N = 42, 7)
		(5%, partial*)	(5%, partial**)
Induction of cell rounding	*fng-Gal4* (*Tub-Gal80* ^*TS*^) > *sterile 20-like kinase*	0% (N = 34, 7)	0% (N = 34, 7)
		(12%, partial*)	(18%, partial**)
	*fng-Gal4* (*Tub-Gal80* ^*TS*^) > *shotgun.IR*	0% (N = 37, 6)	0% (N = 37, 6)
		(8%, partial*)	(14%, partial**)
Disruption of centrosome***	*centrosomin* ^*HK21*^ homozygote	6% (N = 36, 6)	6% (N = 36, 6)
		(11%, partial*)	(8%, partial**)
	*Sas-4* ^*S2214*^ homozygote	9% (N = 34, 7)	9% (N = 34, 7)
		(18%, partial*)	(29%, partial**)
Activation of contractile ring formation	*fng-Gal4* (*Tub-Gal80* ^*TS*^) > *pebble*	0% (N = 34, 6)	44% (N = 34, 6)
		(56%, partial*)	(41%, partial**)
	*fng-Gal4* (*Tub-Gal80* ^*TS*^) > *sqh* ^*D20.D21*^	6% (N = 33, 6)	39% (N = 33, 6)
		(64%, partial*)	(48%, partial**)
	*sqh-GFP*	11% (N = 36, 6)	14% (N = 36, 6)
		(47%, partial*)	(53%, partial**)
	*Septin-2-GFP*	3% (N = 36, 7)	14% (N = 36, 7)
		(22%, partial*)	(42%, partial**)

Numbers of cells and tissues (pairs of lobes) observed are shown as (N = cells, tissues).

*A phenotype class with partial assembly of central spindle-like structure.

**A phenotype class with partial accumulation of F-actin accompanied by furrow progression.

***Very low frequency of cells shows obvious misorientation of spindle axis.

Mis-assembly of the central spindle and incomplete formation of the contractile ring strongly induced skipping of cytokinesis during binucleation. The formation of the actin-contractile ring requires sufficient activation of Rho GTPase at the division plane [8]. To activate Rho GTPase encoded by Rho1, we temporarily elevated the level of the RhoGEF pebble around the binucleation stage. Pebble activates a Rho1 signaling cascade that phosphorylates MRLC (a regulatory light chain of non-muscle myosin II) encoded by *spaghetti squash* (*sqh*) to form the contractile ring [5,7,8]. Overexpression of *pebble* resulted in F-actin accumulation at the cleavage furrow, a sign of contractile ring formation, and in furrow ingression during the binucleation stage (Figure 2K–O and K’–O’) (Table 1). We also found that overexpression of the activated form of *sqh* (*sqh*^*D20.D21*^) induced the formation of the contractile ring (Additional file 1: Figure S4I) (Table 1). Furthermore, we tested a moderate overexpression of wild-type *sqh* or *Septin-2* to see whether cells in the binucleation stage have a latent ability to create a contractile ring. Since the septin proteins are contractile ring components that act together with actomyosin and microtubules [26], we could easily see contractile ring formation in the telophase cells (Additional file 1: Figure S4J–M) (Table 1). These results indicate that the level of central spindle assembly in accessory gland cells in telophase was too low to sufficiently activate Rho1 signaling for cytokinesis.

### Mud regulates central spindle assembly, spindle orientation and cell rounding during binucleation

**Figure 4:**
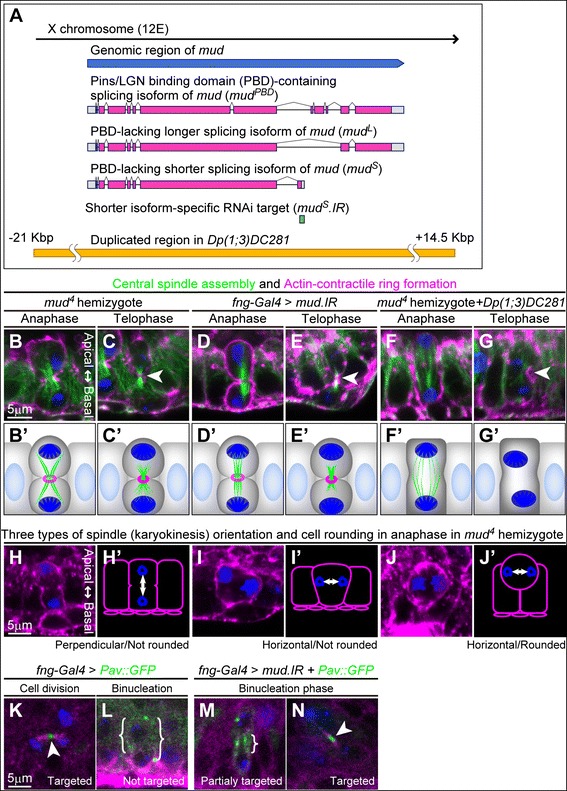
**Loss-of-function for** ***mud*** **erases various characteristics of binucleation. (A)** Schematic diagram of the *mud* transcriptional unit and three representative splicing variants of *mud* (coding regions are in magenta). Regions corresponding to *mud* ^*S*^ *.IR* (green) and chromosomal duplication in *Dp(1;3)DC281* (yellow) are also shown. **(B–G)** Cross-sectional views of main cells in late anaphase **(B, D and F)** and telophase **(C, E and G)** during the binucleation stage in mutants hemizygous for *mud* ^*4*^ **(B and C)**, in knockdown for *mud* **(D and E)** and in mutants hemizygous for *mud* ^*4*^ rescued by one copy of *Dp(1;3)DC281* **(F and G)**. Cells are labeled with phalloidin (magenta), anti-α-Tub antibody (green), and anti-P-H3 antibody (blue). Arrowheads in **(C, E and G)** indicate equatorial planes. Scale bar in **(B)**, 5 μm, is applicable to **(B–G)**. **(B’–G’)** Schematic diagrams of **(B and G)**. **(H–J)** Three representative types of spindle orientation and cell shapes (bottom). Cross-sectional views of main cells in late anaphase during the binucleation stage in mutants hemizygous for *mud* ^*4*^ are shown. Cells are labeled with phalloidin (magenta) and anti-P-H3 antibody (blue). Scale bar in **(H)**, 5 μm, is applicable to **(H–J)**. **(H’–J’)** Schematic diagrams of **(H–J)**. **(K–N)** Main cells expressing Pav::GFP plus ends marker in telophase in wild-type **(K,L)** and *mud*-knockdown **(M,N)** cells. Cell division stage in early pupa (K, *en fac*e view) and binucleation stage in mid-pupa (**L–N**, cross sectional views) are shown. Cells are labeled with anti-α-Tub antibody (magenta), Pav::GFP fluorescence with anti-GFP antibody (green) and anti-P-H3 antibody (blue). Arrowheads in **(K and N)** and curly brackets in **(L and M)** indicate the localization of Pav::GFP on microtubules. Scale bar in **(K)**, 5 μm, is applicable to **(K–N)**.

The insufficient activity of Rho1 signaling in binucleation is thought to be due to the insufficient assembly of the central spindle from anaphase to telophase, so we hypothesized that factors that repressed the central spindle assembly would be key regulators in skipping cytokinesis. Moreover, as stated above, the reduced central spindle assembly during binucleation should be accompanied by a non-round cell shape and orientation of the mitotic spindle along the apico-basal axis. Thus we examined candidate factors – such as mitotic kinesins (*kinesin-like protein at 10A, kinesin-like protein at 61 F, Pavarotti* and *no distributive disjunction*) [4], microtubule-associated proteins (*chromosome bows* and *Eb1*) [4], microtubule-severing proteins (*katanin 60*, *spastin*) [27], Par proteins (*bazooka*, *par-1*) [28] and spindle orientation proteins (*rapsynoid*, *G protein αi subunit* and *mushroom body defect* (*mud*)) [29-31], with regard to their effects on cellular phenotypes of accessory gland cell binucleation. We found that loss of *mud* disrupted normal binucleation phenotypes, including reduced central spindle assembly and other features of cell morphology during binucleation (Figure 4) (Tables 2 and 3). *mud* encodes the *Drosophila* homolog of NuMA, which is associated with microtubules and plays a role in microtubule polymerization and determination of the spindle orientation [29-31]. In *mud*^*4*^ hemizygotes and *mud*-knockdown cells, abnormally clear central spindle assembly and contractile ring formation were seen even in the binucleation stage (Figure 4B–E and B’–E’ compared with Figure 2I, J, I’ and J’) (Table 2 and Table 3). Loss of *mud* was also associated with other morphological defects, such as abnormal spindle orientation and cell rounding (Figure 4H–J and H’–J’ compared with Figure 2H and H’) (Table 2 and Table 3). We thus confirmed that one copy of a chromosomal duplication encompassing the *mud* gene region (Figure 4A) rescued these defects (Figure 4F, G, F’ and G’) (Table 2). These results suggest that Mud contributes to various cell morphologies during binucleation. We considered its repression of the central spindle assembly to be a major cause of binucleation because in *mud*^*4*^ hemizygotes, we frequently observed cytokinesis progression even when neither horizontal spindle orientation nor cell rounding was observed (Figure 4B–E and B’–E’) (Additional file 2: Table S1).

**Table 2:** **Ability of Mud variants to rescue binucleation defects in** ***mud*** **mutants**

**Genotype**		**Frequencies of phenotypes in mitotic behavior during binucleation**			
		**Central spindle assembly**	**Contractile ring formation**	**Cell rounding**	Abnormal spindle orientation***
Wild-type (control)		0% (N = 42, 7)	0% (N = 42, 7)	0% (N = 49, 5)	6% (N = 49, 5)
		(5%, partial*)	(5%, partial**)		
*mud* ^*4*^ hemizygote (*mud* ^*4*^/*Y*) + rescue construct (RC)	No RC	82% (N = 40, 9)	87% (N = 40, 9)	30% (N = 40, 8)	40% (N = 40, 8)
		(15%, partial*)	(13%, partial**)		
	RC = *DC281*	0% (N = 30, 4)	0% (N = 30, 4)	8% (N = 38, 4)	5% (N = 38, 4)
		(40%, partial*)	(57%, partial**)		
	RC = *mud* ^*PBD*^	63% (N = 30, 4)	67% (N = 30, 4)	37% (N = 30, 4)	17% (N = 30, 4)
		(33%, partial*)	(33%, partial*)		
	RC = *mud* ^*L*^	30% (N = 43, 9)	30% (N = 43, 9)	12% (N = 41, 7)	44% (N = 41, 7)
		(51%, partial*)	(56%, partial**)		
	RC = *mud* ^*S*^	13% (N = 40, 8)	18% (N = 40, 8)	34% (N = 44, 7)	20% (N = 44, 7)
		(45%, partial*)	(33%, partial**)		

Numbers of cells and tissues (pairs of lobes) observed are shown as (N = cells, tissues).

*A phenotype class with partial assembly of central spindle-like structure.

**A phenotype class with partial accumulation of F-actin accompanied by furrow progression.

***Spindle orientated horizontally (0° ± 45°) rather than vertically in metaphase.

*DC281*: Chromosomal duplication encompassing the *mud* gene region.

**Table 3:** **Effects of knockdown for** ***mud*** **on binucleation and asymmetric cell division (asym. cell div.)**

**RNAi target**	**Frequencies of phenotypes in mitotic behaviors during**				
	**binucleation**				**asym. cell div.**
	**Central spindle assembly**	**Contractile ring formation**	**Cell rounding**	Abnormal spindle orientation***	**Multi-bristle**
None (*fng-Gal4*)	0% (N = 35, 6)	0% (N = 35, 6)	5% (N = 40, 6)	8% (N = 40, 6)	0% (N^W^ = 60)
	(0%, partial*)	(3%, partial**)			
*mud.IR*	80% (N = 35, 7)	86% (N = 35, 7)	28% (N = 40, 5)	35% (N = 40, 5)	85% (N^W^ = 40)
	(20%, partial*)	(14%, partial**)			
*mud* ^*S*^ *.IR + Dcr2*	27% (N = 37, 5)	29% (N = 37, 5)	5% (N = 42, 5)	31% (N = 42, 5)	0% (N^W^ = 40)
	(51%, partial*)	(41%, partial**)			

Numbers of cells and tissues (pairs of lobes) observed are shown as (N = cells, tissues).

Numbers of adult wings observed are shown as (N^W^ = wings).

*A phenotype class with partial assembly of central spindle-like structure.

**A phenotype class with partial accumulation of F-actin accompanied by furrow progression.

***Spindle orientated horizontally (0° ± 45°) rather than vertically in metaphase.

### Mud represses development of central spindle during binucleation

We showed that Mud repressed central spindle assembly and led to cytokinesis skipping. We made further observations of the central spindle assembly in wild-type and *mud*-knockdown cells in the binucleation stage. In particular, we used Pav::GFP (GFP-fusion of Pavarotti, a centralspindlin component) as a marker for the plus ends of the microtubules in the mitotic spindle [32,33]. Regarding the control results, during the standard cell division at the early pupal stage, Pav::GFP was localized to the midzone of the central spindle at telophase, as reported previously (Figure 4K). During binucleation, in contrast, we never observed Pav::GFP-accumulated microtubules around the cell equator region (Figure 4L). Detailed observation of Pav::GFP accumulation revealed that the impairment of central spindle assembly during binucleation was not due to impaired initiation of microtubule assembly. That is because the Pav::GFP accumulation around cell equator at anaphase was similarly observed in the case of dividing cells, implying that the central spindle precursor could develop at early anaphase (Additional file 1: Figure S5F and F’ compared with A and A’). But during binucleation, impaired microtubule bundling or destabilization of the microtubules was apparent at late anaphase (Additional file 1: Figure S5G–J and G’–J’ compared with C–E and C’–E’). As a result, during binucleation the development of microtubules was insufficient for their interdigitation at the spindle midzone. We also observed that the contractile ring component Peanut (Pnut), a Septin family protein, was localized around the cleavage furrow at anaphase but diffused during telophase (Additional file 1: Figure S6D–F and D’–F’ compared with A–C and A’–C’). These results imply that the contractile ring begins to form during anaphase and is degraded during telophase.

We also checked whether the loss of *mud* affected the localization of Pav::GFP during binucleation. In *mud-*knockdown cells, the localization of Pav::GFP was restricted to the midzone of the central spindle (Figure 4N). We also found that this localization was correlated with the central spindle formation. Cells with partially assembled central spindles showed a partial localization of Pav::GFP at the midzone (Figure 4M), whereas cells with strongly assembled central spindles showed a clear localization (Figure 4N). These results suggest that Mud represses microtubule polymerization so much that microtubule filaments are not targeted around the cell equator.

### Isoform-specific functions of Mud regulate various traits in binucleation

Although Mud is known to promote polymerization of microtubules [34], our results showed that Mud may be a negative regulator of spindle formation during the binucleation process. The above results also indicate that unknown functions of Mud regulate central spindle assembly, spindle orientation, and cell rounding. Interestingly, *mud* generates various splicing variants that have different C-termini (Additional file 1: Figure S3A and referred to in http://flybase.org). To understand the relationship between these unknown functions of Mud and its structural isoforms that had not fully been analyzed, we compared the functions of three different splicing variants (Figure 4A) (Additional file 1: Figure S7): a Pins binding domain (PBD)-containing isoform (Mud^PBD^), a PBD-lacking longer isoform (Mud^L^) and a PBD-lacking shorter isoform (Mud^S^). Mud^PBD^ is well-known major variant that regulates spindle polarity in a Pins/LGN-dependent manner and promotes microtubule polymerization [29-31,34]. Mud^L^ and Mud^S^, both of which lack the Pins/LGN-binding domain (Figure 4A) (Additional file 1: Figure S7), have not yet been examined and their functions are unclear. We thus expected that *mud*^*L*^ or *mud*^*S*^ would exhibit novel functions that regulate central spindle assembly and spindle orientation during binucleation.

**Figure 5:**
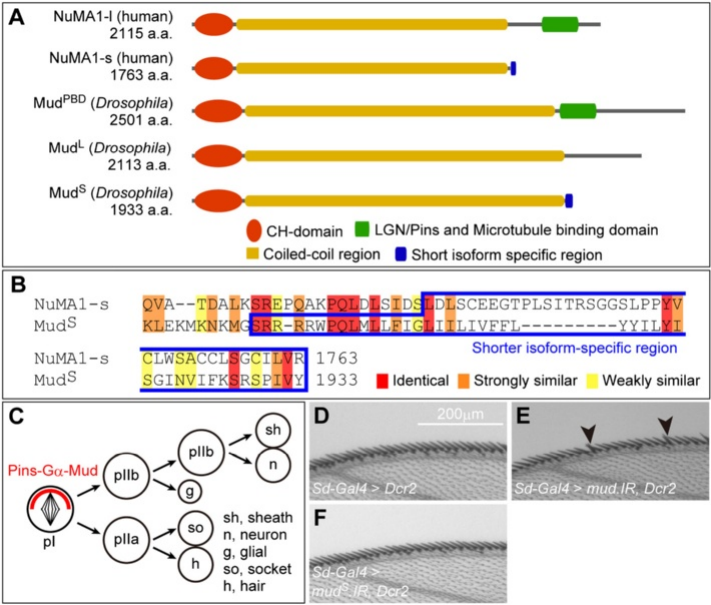
***mud*** ^***S***^ **is not required for the spindle orientation during asymmetric cell division. (A)** Schematic diagram of molecular structures of human NuMA1 and *Drosophila* Mud. Shared domains are as indicated at the bottom. **(B)** Amino acid sequence alignment of the C-terminal regions of NuMA1-s and Mud^S^. The blue box indicates short isoform- specific regions in NuMA1-s (amino acids 1701–1763) and Mud^S^ (amino acids 1880–1933). Red, orange and yellow overlays indicate similarities as indicated at the bottom. **(C)** Schematic diagram of SOP lineage in *Drosophila* wing margin. Cells indicated at the bottom right are produced in this lineage. Mud forms a complex with Gα and Pins that is localized asymmetrically (red crescent). **(D–F)** Bristles on adult anterior wing margin in wild-type **(D)**, in knockdown of all *mud* isoforms **(E)**, and in knockdown specific to *mud* ^*S*^ **(F)**. Arrowheads in **(E)** indicate multi-bristle phenotypes.

**Figure 6:**
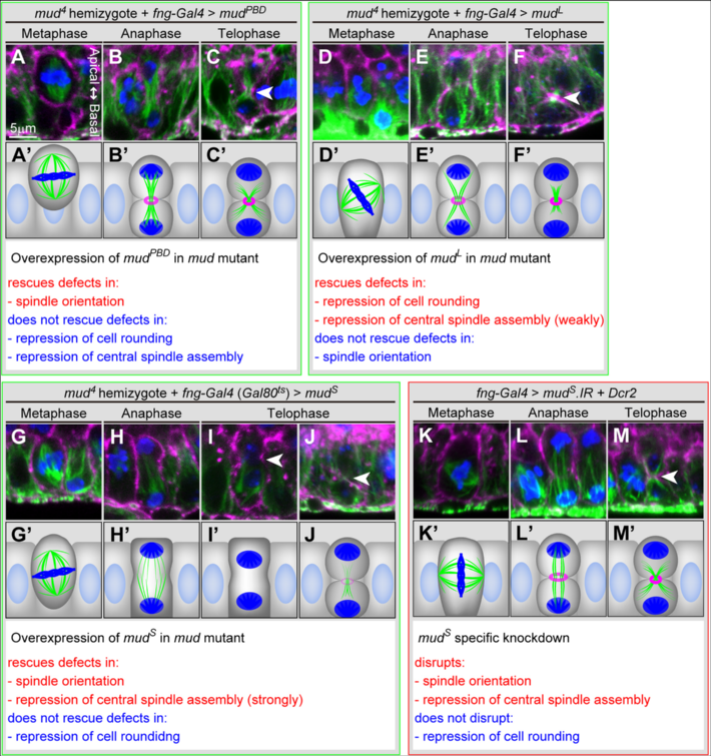
**Three types of** ***mud*** **splicing isoforms differently regulate morphologies of binucleating cells. (A–J)** Rescue of *mud* mutant phenotypes by each *mud* isoform. Cross-sectional views of main cells in metaphase **(A, D and G)**, late anaphase **(B, E and H)** and telophase **(C, F, I and J)** during the binucleation stage in mutants hemizygous for *mud* ^*4*^ with overexpression of *FLAG::mud* ^*PBD*^ **(A–C)**, *FLAG::mud* ^*L*^ **(D–F)** or *FLAG::mud* ^*S*^ **(G–J)**. Cells are labeled with phalloidin (magenta), anti-α-Tub antibody (green) and anti-P-H3 antibody (blue). The cell in **(I)** shows neither a central spindle assembly nor furrow progression. The cell in **(J)** shows furrow progression but no central spindle assembly. Arrowheads in **(C, F, I and J)** indicate equatorial planes. Scale bar in **(A)**, 5 μm, is applicable to **(A–J)**. **(A’–J’)** Schematic diagrams of **(A–J)**. Cross-sectional views of *mud* ^*S*^- knockdown main cells in metaphase **(K)**, late anaphase **(L)** and telophase **(M)** during the binucleation stage. Cells are labeled with phalloidin (magenta), anti-α-Tub antibody (green) and anti-P-H3 antibody (blue). Arrowhead in **(M)** indicates an equatorial plane. Scale bar in **(A)**, 5 μm, is applicable to **(K–M)**. **(K’–M’)** Schematic diagrams of **(K–M)**. Effects on cell morphologies in each of the three rescued genotypes (**(A–C)**: *mud* ^*4*^ hemizygotes rescued by *mud* ^*PBD*^, **(D–F)**: *mud* ^*4*^ hemizygotes rescued by *mud* ^*L*^, **(G–J)**: *mud* ^*4*^ hemizygote rescued by *mud* ^*S*^) and in *mud* ^*S*^-knockdown cells **(K–M)** are listed under each set of diagrams.

To identify isoform-dependent functions of Mud, we tested the ability of Mud^PBD^, Mud^L^ and Mud^S^ to rescue the various defects in binucleation in *mud* hemizygotes. Overexpression of *mud*^*PBD*^ (Additional file 1: Figure S7 and Figure S8) rescued only their abnormal spindle orientation (Figure 6A–C and A’–C’), reducing the frequency of abnormal spindle orientation from 40% in *mud*^*4*^/*Y* to 17% in *mud*^*4*^/*Y + mud*^*PBD*^ (Table 2). Overexpression of *mud*^*S*^ (Additional file 1: Figure S7 and Figure S8) rescued their abnormally enhanced central spindle assembly (Figure 6H–J and H’–J’), reducing the frequency of central spindle assembly from 82% in *mud*^*4*^/*Y* to 13% in *mud*^*4*^/*Y + mud*^*S*^ (Table 2). *mud*^*S*^ also partially rescued the abnormal spindle orientation (Figure 6G and G’), reducing the frequency of abnormal spindle orientation from 40% in *mud*^*4*^/*Y* to 20% in *mud*^*4*^/*Y + mud*^*S*^ (Table 2). In contrast, the overexpression of *mud*^*L*^ (Additional file 1: Figure S7 and Figure S8) effectively rescued the cell rounding phenotype found in *mud* hemizygotes (Figure 6D and D’), reducing the frequency of cell rounding from 30% in *mud*^*4*^/*Y* to 12% in *mud*^*4*^/*Y + mud*^*L*^ (Table 2). *mud*^*L*^ also partially rescued the abnormally enhanced assembly of central spindle (Figure 6E, E’, F and F’), reducing the frequency of central spindle assembly from 82% in *mud*^*4*^/*Y* to 30% in *mud*^*4*^/*Y + mud*^*L*^ (Table 2), but its ability was obviously less than that of *mud*^*S*^ (compare the reduction to 30% by *mud*^*4*^/*Y + mud*^*L*^ with the reduction to 13% by *mud*^*4*^/*Y + mud*^*S*^). These results imply that Mud has the following isoform-dependent functions during binucleation: Mud^L^ represses cell rounding and weakly represses central spindle assembly, Mud^PBD^ and Mud^S^ each play a role in orienting the spindle axis along the apico-basal axis, and Mud^S^ also strongly represses central spindle assembly.

**Figure 7:**
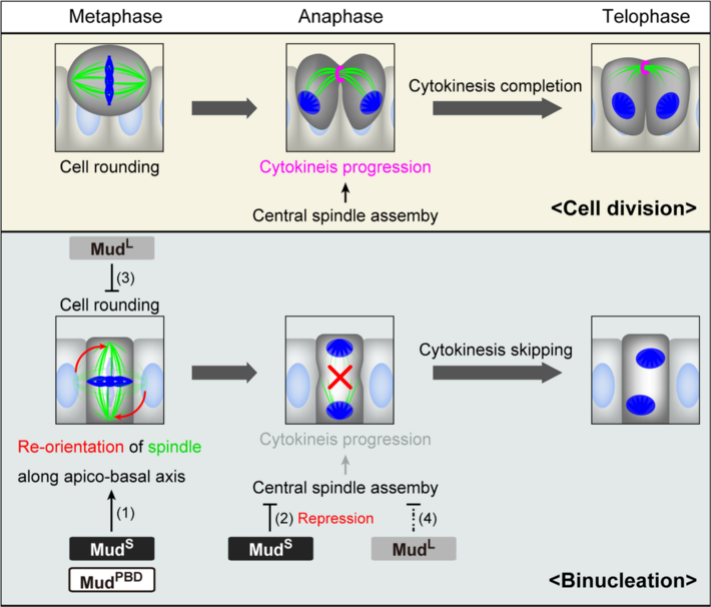
**Model for binucleation and isoform-specific functions of Mud.** Schematic diagrams of cell division and binucleation are shown. In binucleation, Mud^S^ changes mitosis from cell division to binucleation by (1) reorienting the mitotic spindle from horizontal to vertical along the apico-basal axis and (2) repressing assembly of the central spindle. Mud^PBD^ is also required at this time for orienting the spindle along the apico-basal axis. Mud^L^, in contrast, (3) represses mitotic cell rounding and may assist in the process of cytokinesis skipping by (4) partially repressing the assembly of the central spindle.

### Mud^S^ orients mitotic spindle along the apico-basal polarity and inhibits cytokinesis

The above overexpression results suggest that Mud^S^ contributes to cytokinesis skipping during binucleation more than Mud^L^ does, but the functions of Mud^S^ during *Drosophila* development have not yet been reported. We therefore tried to determine whether endogenously expressed *mud*^*S*^ actually regulates binucleation in the accessory gland. To do so, we performed a *mud*^*S*^-specific knockdown (Figure 4A and Figure 6H–K and I’–K’) (Additional file 1: Figure S7 and Figure S8) and found that the *mud*^*S*^-knockdown cells showed abnormally enhanced assembly of the central spindle (Figure 6L, L’, M and M’) and abnormal orientation of the mitotic spindle (Figure 6K and K’) (Table 3). These results strongly suggest that Mud^S^ represses central spindle assembly and orients the spindle axis vertically but does not regulate cell rounding.

Mud^PBD^ is known to regulate spindle orientation during asymmetric cell division, so we tried to determine whether the spindle orientation-regulating function of Mud^S^ during binucleation is independent of that of Mud^PBD^. We tried to do that by determining whether a knockdown of Mud^S^ affected asymmetric cell division in the sensory organ precursor cells (SOPs) on the adult wing margins (Figure 5C). The spindle orientation of this asymmetric cell division is known to be regulated by Mud^PBD^ in a Pins- and Gαi-dependent manner, and loss of this function results in a multi-bristle phenotype on the adult wing (Figure 5C). In fact, the knockdown of all Mud isoforms caused abnormal spindle orientation and the multi-bristle phenotype (arrowheads in Figure 5E compared with D) (Table 3) [29,35,36]. The knockdown of *mud*^*S*^ alone, in contrast, did not induce the multi-bristle phenotype (Figure 5F) (Table 3), although it effectively caused spindle orientation changes during binucleation (Figure 6I and I’) (Table 3). These results suggest that even though Mud^S^ regulates spindle orientation during binucleation, it is not involved in the spindle orientation during asymmetric cell division of SOPs.

**Figure 8:**
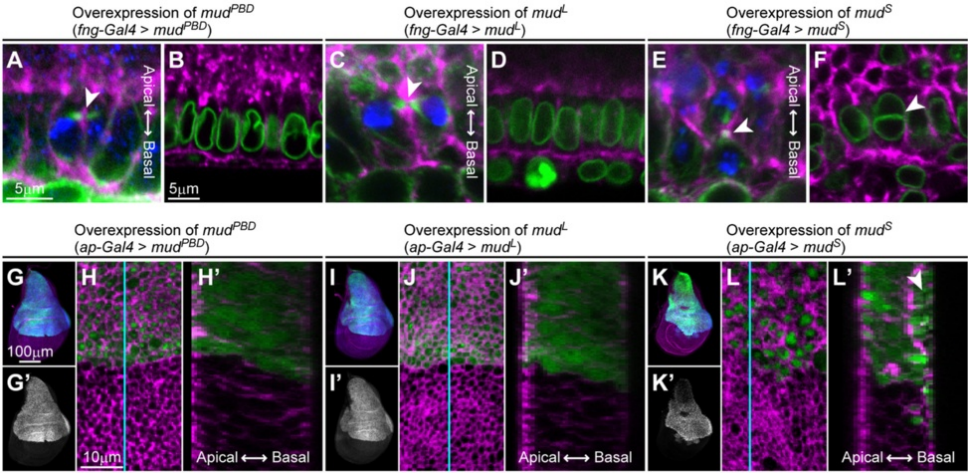
**Overexpression of** ***mud*** ^***S***^ **is sufficient for converting dividing cells into binucleating ones. (A–F)** Cross-sectional views of main cells in early pupal proliferating stage in which *mud* ^*PBD*^ **(A and B)**, *mud* ^*L*^ **(C and D)** or *mud* ^*S*^ **(E and F)** are overexpressed. Cells in telophase **(A, C and E)** or interphase **(B, D and F)** are shown. Cells are labeled with phalloidin (magenta), anti-α-Tub antibody **(green in A, C and E)**, anti-LamDm_0_ antibody **(green in B, D and F)** and anti-P-H3 antibody (blue). Arrowheads in **(A, C and E)** indicate equatorial planes. The arrowhead in **(F)** indicates a binucleate cell. Scale bar in **(A)**, 5 μm, is applicable to **(A, C and E)**. Scale bar in **(B)**, 5 μm, is applicable to **(B, D and F)**. **(G, G’, I, I’, K and K’)** Wing imaginal discs in which *FLAG::mud* ^*PBD*^ **(G)** *, FLAG::mud* ^*L*^ **(I)** or *FLAG::mud* ^*S*^ **(K)** is induced in their dorsal compartment (labeled with GFP). Cells are labeled with phalloidin (magenta), GFP (green) and anti-FLAG antibody (blue). Gray-scale images in **(G’, I’ and K’)** are of the blue channels in **(G, I and K)**. Scale bar in **(G)**, 100 μm, is applicable to **(G, G’, I, I’, K and K’)**. **(H, J and L)** Magnified views of **(G, I and K)** around the dorso-ventral boundaries. Magenta and green channels are shown. Cells with expression of *mud* ^*S*^ were enlarged in volume (L), possibly as a result of cytokinesis defects. **(H’, J’ and L’)** Cross-sectional images reconstructed by using a stack of confocal sections at cyan lines in **(H, I and L)**. The epithelium with expression of *mud* ^*S*^ has an abnormally folded or layered structure (**arrowhead in L’**). Scale bar in **(H)**, 10 μm, is applicable to **(H, H’, J, J’, L and L’)**.

As shown above, Mud^S^ seems to convert the mitotic morphologies from the cell-division type (horizontal spindle orientation and cytokinesis progression) to the binucleation type (vertical spindle orientation and cytokinesis skipping) (Figure 6) (Table 2 and Table 3). To examine this hypothesis, we tested the ability of Mud^S^ to convert the mitotic morphologies of cells in the early pupal accessory gland primordia, in which standard cell division occurs. The results showed that the spindle orientation changed from horizontal to vertical (Figure 8E compared with Figure 2D). Furthermore, binucleate cells appeared with some frequency (arrowhead in Figure 8F). This effect of overexpression of *mud*^*S*^ on cell morphologies can be seen not only in the accessory gland but also in the wing imaginal disc (Figure 8K, K’, L and L’). Overexpression of *mud*^*PBD*^ or *mud*^*L*^, in contrast, affected neither spindle orientation nor cytokinesis (Figure 8A–D, G–J and G’–J’). These results suggest that Mud^S^ can convert mitotic morphologies from the cell-division type to the binucleation type.

## Discussion

### Regulation of M-phase entry in binucleation of *Drosophila* male accessory gland

We characterized the final M-phase entry that contributed to binucleation of cells in the *Drosophila* male accessory gland (Figure 1D) (Additional file 1: Figure S1). We showed that the entry into the binucleation stage took place with a two-step cell cycle transition: cell cycle arrest for 5 hours and subsequent synchronous entry into the M phase (Figure 1D) (Additional file 1: Figure S1). Thus, standard cell division and binucleation are separated by a 5-hour interval of cell cycle arrest (Additional file 1: Figure 1D) (Additional file 1: Figure S1). This may indicate that standard mitosis and binucleation have very different regulations of the cell cycle and cytokinesis. In fact, although in most tissues in *Drosophila* standard mitosis can occur normally without the spindle checkpoint [37], we previously demonstrated that the knockdown of *mad2*, a spindle checkpoint component, frequently causes a defect in chromosomal segregation in accessory gland cells [16]. This is consistent with our proposal that binucleation is regulated by a system different from the one regulating standard mitosis.

### Morphological features of mitotic cells during binucleation

We identified three morphological features characteristic of binucleation: the non-round shape of mitotic cells, the apico-basal orientation of the mitotic spindle and the poor assembly of the central spindle (Figure 2F–J and F’–J’ compared with A–E and A’–E’). The reduction of central spindle assembly could directly repress cytokinesis, but we did not find the significance of the vertical orientation of the spindle or the non-round shape of the cell. We can explain it as follows. If most cells of the epithelium synchronously enter the M phase with the cell rounding phenotype, the stability of the monolayer epithelium may be severely disrupted. In fact, we observed a severe defect in epithelial stability after the binucleation stage in a *mud* mutant in which the cells were rounded during the binucleation stage (data not shown). In addition, if the spindles in columnar cells are horizontally oriented, they will be less stable than they would be if they were vertically oriented because a spindle is aligned more stably along a longer axis than a shorter one [38]. Vertical orientation of the spindle and lack of cell rounding may thus be appropriate for synchronous binucleation of columnar cells, but these morphological features do not directly regulate cytokinesis skipping.

### Mechanisms by which Mud regulates central spindle assembly, spindle orientation, and cell rounding during binucleation

We propose that Mud is a key factor in regulating binucleation. We demonstrated that Mud functions in a way that represses central spindle assembly, orients the mitotic spindle along the apico-basal axis and inhibits mitotic cell rounding during mitosis (Figure 7). We found a clue as to how Mud represses the central spindle assembly. In standard cell division, during late anaphase the microtubules of the central spindle are polymerized in order to target their plus ends at the cell equator [32,33], and we confirmed this in our experiments in which the plus ends were labeled with Pav::GFP (Figure 4K). During binucleation, in contrast, the Pav::GFP marker did not sufficiently target the cell equator (Figure 4L). This implies that the polymerization of the microtubules of the central spindle is insufficient to target them at the cell equator, and the central spindle therefore does not develop completely. We also showed that the Mud is needed in order to repress the growth of microtubules. In fact, the knockdown of *mud* promoted the growth of microtubules that targeted their plus ends at the cell equator even in the binucleation stage (Figure 4M and N). However, the underlying molecular mechanisms of Mud in repressing polymerization of microtubules remain unclear. Moreover, the question of how Mud regulates the mitotic spindle orientation along the apico-basal axis and how it inhibits mitotic cell rounding are also unclear. The logical next step will be to relate the isoform of each Mud to various effector molecules regulating the orientation and the rounding.

### Alternative splicing of *mud*/*NuMA* produces three types of structurally different proteins

We showed that the three types of alternative splicing products Mud^PBD^, Mud^L^ and Mud^S^ have distinct functions from one another (Figure 6, Figure 5, Figure 8 and Figure 7). It is known that the *mud* gene produces four isoforms (RH, RI, RJ and RL) that contain the Pins/LGN binding domain (PBD) and three isoforms (RF, RG and RK) that do not contain it (Figure 5A) (Additional file 1: Figure S7) (http://flybase.org). Moreover, the three PBD-lacking isoforms are structurally classified into the following two types: a longer isoform (RF) that simply skips the PBD-encoding exons by alternative splicing, and two shorter isoforms (RG and RK) that contain a shorter isoform-specific exon instead of the PBD-encoding exons (Figure 5A) (Additional file 1: Figure S7).

At least one of the PBD-containing isoforms (RL) is functional and known to regulate the spindle orientation in a Pins/LGN-dependent manner in asymmetric cell division [29-31]. In contrast, although *mud*^*L*^ and *mud*^*S*^ are actually transcribed [34] there is no evidence that Mud^L^ and Mud^S^ are functional and have Pins/LGN-independent functions. As in the *Drosophila* gene *mud*, splicing variants also occur in human *NuMA1*, and these variants encode a longer isoform (NuMA1-l), a shorter isoform (NuMA1-s) and a medium isoform (NuMA1-m) [39]. Although the functions of NuMA1-m and NuMA1-s are unclear, NuMA1-l has an LGN-binding domain in the C-terminal region and determines the spindle polarity in an LGN-dependent manner, the same as in the case of *Drosophila* Mud^PBD^ (Figure 5A) [40]. NuMA1-m and NuMA1-s, in contrast, like *Drosophila* Mud^L^ and Mud^S^ do not have an LGN-binding domain in the C-terminal region (Figure 5A). Interestingly, we found sequence similarities between human NuMA1-s and *Drosophila* Mud^S^ in their C-terminal domains, including the shorter isoform-specific regions (Figure 5B). These similarities suggest that Mud^S^ in *Drosophila* functions similarly to NuMA1-s in humans.

### Isoform-dependent functions of Mud mediate various morphological changes of binucleating cells

We showed that the functions of *mud*^*PBD*^, *mud*^*L*^ and *mud*^*S*^ are independent during binucleation. The repression of mitotic cell rounding was a Mud^L^-specific function. In contrast, changing the orientation of the mitotic spindle along the apico-basal axis was controlled by both Mud^PBD^ and Mud^S^ (Figure 6 and Figure 7) (Table 2 and Table 3). Mud^PBD^ was previously reported to be required for the spindle orientation during asymmetric cell division [29,35,36]. We showed, however, that Mud^S^ is not associated with the spindle orientation during asymmetric cell division (Figure 5F). On the other hand, the overexpression of *mud*^*S*^ but not *mud*^*PBD*^ reoriented the spindle along the apico-basal axis in dividing cells (Figure 8A and E). These results suggest that Mud^PBD^ and Mud^S^ regulate the spindle orientation independently.

The function repressing central spindle assembly during binucleation was also shared by Mud^L^ and Mud^S^, but we showed that Mud^S^ contributed the most to repressing spindle assembly. In fact, the overexpression of *mud*^*S*^ effectively rescued the *mud*^*4*^ mutant phenotype, the abnormally enhanced assembly of the central spindle (Figure 6I and J) (Table 2). In addition, like the *mud*^*4*^ mutant, a *mud*^*S*^-specific knockdown abnormally enhanced central spindle assembly in the binucleation stage (Figure 6L and M) (Table 3). Mud^L^, in contrast, only partially repressed the central spindle assembly during binucleation (Figure 6F) (Table 2). Moreover, overexpression of *mud*^*S*^, but not *mud*^*L*^, inhibited cytokinesis to produce binucleate cells in dividing cells such as the early pupal accessory gland cells (Figure 8D and F) and the larval wing disc cells (Figure 8J and L). This also suggests that *mud*^*S*^ mainly contributes to the repression of central spindle assembly (Figure 7).

## Conclusions

We described the binucleation event of the *Drosophila* male accessory gland during pupal development and analyzed the cellular mechanisms regulating this binucleation. We characterized a unique cell cycle regulation in the developing accessory gland: the M-phase entry for binucleation occurred synchronously at 55 APF after a cell cycle arrest for 5 hours. We also found that Mud, the *Drosophila* homolog of mammalian NuMA, regulated various features of the binucleating cells, such as a non-round shape, spindle orientation along the apico-basal axis, poor assembly of the central spindle and cytokinesis skipping. It is known that Mud binds Pins to determine the mitotic spindle orientation during the standard cell division or asymmetric cell division [29-31,41,42]. Interestingly, we found atypical functions of Mud that depended on three types of splicing isoforms, each differently regulating the above various features of binucleating cells. We concluded that Mud^PBD^, which is a well-known isoform having a PBD (Figure 5A) (Additional file 1: Figure S7), oriented the spindle along the apico-basal axis. Mud^L^, one of the newly characterized isoforms and simply lacking a PBD, inhibited the mitotic cell rounding and weakly impaired the central spindle assembly (Figure 7). Mud^S^, another newly characterized isoform, containing a shorter isoform-specific domain instead of a PBD-containing domain (Figure 5A) (Additional file 1: Figure S7), oriented the spindle along the apico-basal axis and strongly impaired the central spindle assembly (Figure 7). Importantly, overexpression of Mud^S^ induced an ectopic binucleation even in the cell division stage, whereas overexpression of Mud^PBD^ or Mud^L^ did not (Figure 8). These results suggest that Mud^S^ is an important regulator triggering cytokinesis skipping in binucleation. Abnormal expression of NuMA is known to be correlated with the production of cancer cells in mammals [43]. Our finding of atypical functions of Mud may contribute to the understanding of the relationship between NuMA and tumor progression.

## Methods

### *Drosophila* strains

*Canton-S* and *w*^*1118*^ were used as wild-type strains and the following mutant alleles were used: a functional null allele *centrosomin*^*HK21*^ [25], a strong loss-of-function allele *Sas-4*^*S2214*^ [23], and a strong loss-of-function allele *mud*^*4*^ [34]. *Dp(1:3)DC281* is a chromosome with a duplication of the *mud* gene region [44]. *fng*^*NP5399*^ (*fng-Gal4*, Gal4 Enhancer Trap Insertion Database, http://kyotofly.kit.jp/stocks/GETDB/getdb.html) expresses *Gal4* in the pupal accessory gland epithelial cells and larval-pupal wing disc (data not shown). *AyGal4*, *apterous*^*MD544*^ (*ap-Gal4*), and *Act5C-Gal4* have been described previously [45,46]. *hs-FLP* was used as the source of the FLP recombinase [47]. *Tub-Gal80*^*TS*^ was used for the TARGET system [48]. *UAS-Sterile20-like kinase*, *UAS-pebble* and *UAS-sqh*^*D20.D21*^ express the wild type of *Sterile20-like kinase* [49], the wild type of *pebble* [50] and a constitutively active forms of *sqh* [51]. The strains *103962* (*UAS-shotgun.IR*, VDRC), *mud*^*JF02911*^ (*UAS-mud.IR*, Transgenic RNAi project) [52] and *VALIUM20-mCherry* (*UAS-mCherry.IR*, Transgenic RNAi project at Harvard Medical School) [52] express inverted repeat RNAs (which form hairpin loop double-stranded RNAs) for *shotgun*, *mud*, and *mCherry*. The strains *mus209.ΔNhe::GFP* (*PCNA-GFP*) [53], *Ubi-Cyclin B::GFP* [54], *sqh::GFP* [55] and *Septin2::GFP* [56] have been described previously. *polo*^*CC01326*^ (*polo::GFP*) is a protein trap line of *polo* (FlyTrap, http://cooley.medicine.yale.edu/flytrap/index.aspx#page2) [57].

### Immunostaining and microscopic analysis

The dissected accessory glands were fixed with 4% formaldehyde (Wako) and stained using standard immunostaining protocols. For the DNA staining, the fixed samples were pretreated with RNase (Wako, 0.025 mg/ml) for 15 minutes at 37°C and then stained with propidium iodide (Invitrogen, 1:500). Rhodamine-phalloidin (Invitrogen, 1:40) was used to stain the filamentous actin (F-actin). The following primary antibodies were used: rabbit anti-phospho-histone H3 (P-H3) polyclonal antibody (Millipore, 1:200), mouse anti-α-tubulin (α-Tub) monoclonal antibody (Sigma, 1:50), rat anti-α-tubulin (α-Tub) monoclonal antibody (Millipore, 1:25), rabbit anti-PKCζ (C-20) polyclonal antibody cross-reacting with *Drosophila* aPKC (Santa Cruz, 1:200), mouse anti-coracle (Cora) monoclonal antibody (Developmental Studies Hybridoma Bank, 1:20), mouse anti-lamin Dm_0_ (LamDm_0_) monoclonal antibody (Developmental Studies Hybridoma Bank, 1:40), mouse anti-peanut (Pnut) monoclonal antibody (Developmental Studies Hybridoma Bank, 1:5) and mouse anti-FLAG M2 (FLAG) monoclonal antibody (Sigma, 1:200). The following secondary antibodies were used: Cy3-conjugated donkey anti-mouse IgG (Jackson ImmunoResearch, 1:200), Cy2-conjugated donkey anti-mouse IgG (Jackson ImmunoResearch, 1:200), Cy5-conjugated donkey anti-mouse IgG (Jackson ImmunoResearch, 1:200), Alexa Fluor 488-conjugated donkey anti-rat IgG (Millipore, 1:200) and Cy5-conjugated donkey anti-rabbit IgG (Jackson ImmunoResearch, 1:200). Stained samples were mounted in 50% glycerol/PBS containing 0.25% n-propyl gallate (Wako) and observed with an ECLIPSE TE2000-U with a Digital ECLIPSE C1 and C1Si confocal system (Nikon). Images were processed using EZ-C1 Gold Version 3.70 (Nikon), Adobe Photoshop CS3 Extended (Adobe Systems), and Adobe Illustrator CS3 (Adobe Systems).

### Detection of formation of central spindle and contractile ring

We used microtubule bundles and actin filaments as markers for the central spindle and contractile ring, respectively. In central spindle assembly, microtubule filaments bundle together, forming a large structure that crosses the cell equator. We regarded microtubule-related structures having these features (i.e., bundling and equator-crossing) with fewer microtubule filaments as partially assembled central spindles. However, if the filaments neither associated with each other nor crossed the cell equator, we did not regard the structures as central spindles.

The accumulation of actin filaments in the equatorial region of the cell membrane is usually associated with formation of a cleavage furrow, and we considered a contractile ring to have partially formed if either of these two features was observed.

### Temporal expression of genes using the TARGET system

The TARGET system [48] was used for the temporal expression of genes in the pupal accessory gland epithelium. To restrict the expression of the target genes by activation of Gal80^TS^, flies were reared at a permissive temperature (19°C). To permit moderate expression of the target genes by weak activation of Gal80^TS^, flies were reared at a semi-permissive temperature (26°C).

To fully express *Sterile20-like kinase*, *pebble* and *sqh*^*D20.D21*^ in the accessory gland primordia just before binucleation (Figure 2K–O) (Table 1), pupae reared at 19°C for 110 hours after puparium formation (APF) were incubated at 29°C for 5 hours (genotypes: *w*/*Y*; *Tub-Gal80*^*TS*^/*UAS-Sterile20-like kinase*; *fng-Gal4*/*+*, *w*/*Y*; *Tub-Gal80*^*TS*^/*UAS-shotgun.IR*; *fng-Gal4*/*+*, *w*/*Y*; *Tub-Gal80*^*TS*^/*UAS-pebble*; *fng-Gal4*/*+*, *w*/*Y*; *Tub-Gal80*^*TS*^/*UAS-sqh*^*D20.D21*^; *fng-Gal4*/*+*). To knockdown *shotgun* by expressing *shorgun.IR* in the accessory gland primordia just before binucleation (Additional file 1: Figure S4A and B) (Table 1), pupae reared at 19°C for 80 hours APF were incubated at 29°C for 20 hours (genotypes: *w*/*Y*; *Tub-Gal80*^*TS*^/*UAS-shotgun.IR*; *fng-Gal4*/*+*). To moderately express *mud*^*S*^ in the accessory gland primordia just before binucleation (Figure 6D–G), pupae reared at 19°C for 110 hours APF were incubated at 26°C for 5 hours (genotype: *mud*^*4*^/*Y*; *Tub-Gal80*^*TS*^/*+*; *fng-Gal4*/*UAS-FLAG::mud*^*S*^). To express *mud*^*PBD*^, *mud*^*L*^ and *mud*^*S*^ in the accessory gland primordia in the cell-division stage (Figure 5G–J), pupae reared at 19°C for 40 hours APF were incubated at 29°C for 5 hours (genotypes: *w*/*Y*; *Tub-Gal80*^*TS*^/*+*; *fng-Gal4*/*UAS-FLAG::mud*^*PBD*^, *w*/*Y*; *Tub-Gal80*^*TS*^/*+*; *fng-Gal4*/*UAS-FLAG::mud*^*L*^, *w*/*Y*; *Tub-Gal80*^*TS*^/*+*; *fng-Gal4*/*UAS-FLAG::mud*^*S*^). Pupae were dissected immediately after the target gene inductions described above.

### Construction of plasmids

*pP-Acp70A-Stinger*:

The upstream enhancer of *Acp70A* (-477 to -34) was amplified from the genome DNA of *Canton-S* with a PCR (primer set #1 in Additional file 2: TableS2) and subcloned into T vector *pMD20* (TaKaRa) by using TA cloning. The fragment for the Acp70A enhancer was digested with *Bgl*II and *Not*I and subcloned into *pH-Stinger* (Drosophila Genomics Resource Center (DGRC)) to construct *pP-Acp70A-Stinger*.

*pUAS-FLAG::mud*^*PBD*^*-attB* (see Additional file 1: Figure S7, Figure S8 and Additional file 2: Table S1):

The genomic fragment encompassing three subisoforms of *mud*^*PBD*^ (*mud-RH*, *mud-RI*, and *mud-RL* in Additional file 1: Figure S7) (from just after the start codon to the stop codon) was amplified with a PCR (primer set #2 in Additional file 2: Table S2) from the BAC clone CH322-147E14 (P[acman] Resources) [58] and subcloned into *T vector pMD20* (TaKaRa) by using TA cloning. The forward primer also included the Kozak sequence, start codon and *FLAG* tag sequence. The seventh intron (eighth intron in the case of *mud-RJ*), which included the *mud*^*S*^-specific exon, was removed from the fragment with an inverse PCR (primer set #3 in Additional file 2: Table S2) and ligated to generate a *FLAG*-tagged protein-coding region including *mud-RH*, *mud-RI* and *mud-RL*. This fragment was digested with *Not*I and *Kpn*I and subcloned into *pUASattB* (FlyC31, http://www.flyc31.org/) [59] to generate *pUAS-FLAG::mud*^*PBD*^*-attB.*

*pUAS-FLAG::mud*^*L*^*-attB* (see Additional file 1: Figure S7, Figure S8 and Additional file 2: Table S1):

The genomic fragment encompassing a *mud*^*L*^ (*mud-RF* in Additional file 1: Figure S7) (from just after the start codon to the stop codon) was amplified with PCR (primer set #2 in Additional file 2: Table S2) from the BAC clone CH322-147E14 (P[acman] Resources) [58] and subcloned into *T vector pMD20* (TaKaRa) by using TA cloning. The forward primer also included the Kozak sequence, start codon and *FLAG* tag sequence. The seventh intron, which included the PBD-encoding exons and *mud*^*S*^-specific exon, was removed from the fragment with an inverse PCR (primer set #4 in Additional file 2: Table S2) and ligated to generate a *FLAG*-tagged Mud-RF-coding fragment. This fragment was digested with *Not*I and *Kpn*I and subcloned into *pUASattB* (FlyC31, http://www.flyc31.org/) [59] to generate *pUAS-FLAG::mud*^*L*^*-attB*.

*pUAS-FLAG::mud*^*S*^*-attB* (see Additional file 1: Figure S7, Figure S8 and Additional file 2: Table S2):

The genomic fragment encompassing two subisoforms of *mud*^*S*^ (*mud-RG* and *mud-RK* in Additional file 1: Figure S7) (from just after the start codon to the stop codon) was amplified with a PCR (primer set #5 in Additional files 2: Table S2) from the BAC clone CH322-147E14 (P[acman] Resources) [58] and subcloned into *T vector pMD20* (TaKaRa) by using TA cloning. The forward primer also included the Kozak sequence, start codon and *FLAG* tag sequence. The *FLAG*-tagged fragment, which included both *mud-RG* and *mud-RK*, was digested with *Not*I and *Kpn*I and subcloned into *pUASattB* (FlyC31, http://www.flyc31.org/) [59] to generate *pUAS-FLAG::mud*^*S*^*-attB*.

*pUAS-mud*^*S*^*.IR-attB* (see Additional file 1: Figure S7, Figure S8 and Additional file 2: Table S2):

The genomic fragment including the *mud*^*S*^-specific exon and upstream intron with a splice donor and acceptor (Additional file 1: Figure S7) was amplified with a PCR (primer set #6 in Additional file 2: Table S2) from the genome DNA of *Canton-S* and subcloned into *T vector pMD20* (TaKaRa) by using TA cloning (#1). The *mud*^*S*^-specific exon without the upstream intron was also amplified with a PCR (primer set #7 in Additional file 2: Table S2) from the genome DNA of *Canton-S* and subcloned into *T vector pMD20* (TaKaRa) by using TA cloning (#2). The plasmid #1 was digested with *Not*I and *Xho*I, and the plasmid #2 was digested with *EcoR*I and *Not*I. Both of the digested fragments were combined in a single plasmid *pUASattB* (FlyC31, http://www.flyc31.org/) [59] to generate *pUAS-mud*^*S*^*.IR*.

### Generation of transgenic fly lines

The *pP-Acp70A-Stinger* vector was injected into *y, w* fly lines with standard protocols to generate transgenic lines. The *pUAS-FLAG::mud*^*PBD*^*-attB*, *pUAS-FLAG::mud*^*L*^*-attB*, *pUAS-FLAG::mud*^*S*^*-attB* and *pUAS-mud*^*S*^*.IR-attB* vectors were injected into *y*^*1*^, M{vas-int.Dm}ZH-2A, w*;; *M{3xP3-RFP.attP}ZH-86Fb* or *y*^*1*^, M{vas-int.Dm}ZH-2A, w*; *M{3xP3-RFP.attP'}ZH-51C* with ΦC31-mediated site-specific integration to generate transgenic lines.

### RT-PCR to verify the expression of *UAS*-*mud* isoforms

Total RNA was extracted from third-instar larvae of the following four genotypes: *w*/*Y*;; *hs-Gal4*/*UAS-FLAG::mud*^*PBD*^, *w*/*Y*;; *hs-Gal4*/*UAS-FLAG::mud*^*L*^, *w*/*Y*;; *hs-Gal4*/*UAS-FLAG::mud*^*S*^, and *w*/*Y*;; *hs-Gal4*/+ (negative control). Before the extraction of RNA, larvae were heat-shocked twice at 37°C for 45 minutes and subsequently incubated at 25°C for 2 hours to reach the high level expression of *UAS* targeted genes. The cDNA of each genotype was synthesized using the oligo-dT primer with PrimeScript RT-PCR kit (TaKaRa). Two primer sets (#8 and #9 listed in Additional file 2: Table S2 of the supplementary material) that specifically amplify *FLAG::mud* transgenes but not endogenous *mud* genes (Additional file 1: Figure S8) were used for the PCR.

### Semi-quantitative RT-PCR to verify isoform-specific knockdown by *UAS-mud*^*S*^*.IR*

Total RNA was extracted from third-instar larvae of the following two genotypes: *w*/*Y*; *Act5C-Gal4*/*UAS-mCherry.IR* (control) and *w*/*Y*; *Act5C-Gal4*/+; +/*UAS-mud*^*S*^*.IR* (knockdown for *mud*^*S*^). The cDNA of each genotype was synthesized using the oligo-dT primer with PrimeScript RT-PCR kit (TaKaRa). Expressions of genes were normalized by using *Rpl32* as a reference gene. The three sets of primers #10, #11 and #12 listed in Additional file 2: Table S2 of the supplementary material (Additional file 1: Figure S7) were used for the PCR.

### Animal ethics

All animals used in above genetic experiments were anesthetized with carbon dioxide before each mating. All procedures complied with guidelines of the Animal Ethics Committee of Gakushuin University.

## Additional files

Additional file 1:
**Figures S1, S2, S3, S4, S5, S6, S7 and S8 are included.**
Additional file 2:
**Tables S1** **and** **S2** **are included.**

## Abbreviations

APF: After puparium formation
*mud*: *mushroom body defect*
PBD: Pins binding domain
NuMA: Nuclear mitotic apparatus
RhoGAP: GTPase activating protein for Rho
RhoGEF: Guanine nucleotide exchange factor for Rho GTPase
M phase: Mitotic phase
Acp70A: Accessory gland protein 70A
P-H3: Phospho-histone H3
PCNA: Proliferating cell nuclear antigen
GFP: Green fluorescence protein
*D*E-cadherin: *Drosophila* epithelial cadherin
MRLC: Myosin regulatory light chain
*sqh*: *spaghetti squash*
Par: Partitioning-defective protein
Pav: Pavarotti
Pnut: Peanut
SOP: Sensory organ precursor cell
*fng*: *fringe*
*ap*: *apterous*
*Act5C*: *Actin 5C*
*UAS*: *Upstream activation sequence*
Tub: Tubulin
TARGET: Temporal and regional gene expression targeting
IR: Inverted repeat
aPKC: atypical protein kinase C
Cora: Coracle
LamDm_0_: lamin Dm_0_
