# Supplementary material for: Isoform-specific functions of Mud/NuMA mediate binucleation of Drosophila male accessory gland cells
Source: BMC Dev Biol. 2014 Dec 20;14:46. doi: 10.1186/s12861-014-0046-5 (PMC4300151; doi:10.1186/s12861-014-0046-5)
Supplement: Additional file 1: — Figures S1, S2, S3, S4, S5, S6, S7 and S8 are included. [file 12861_2014_46_MOESM1_ESM.docx]

**
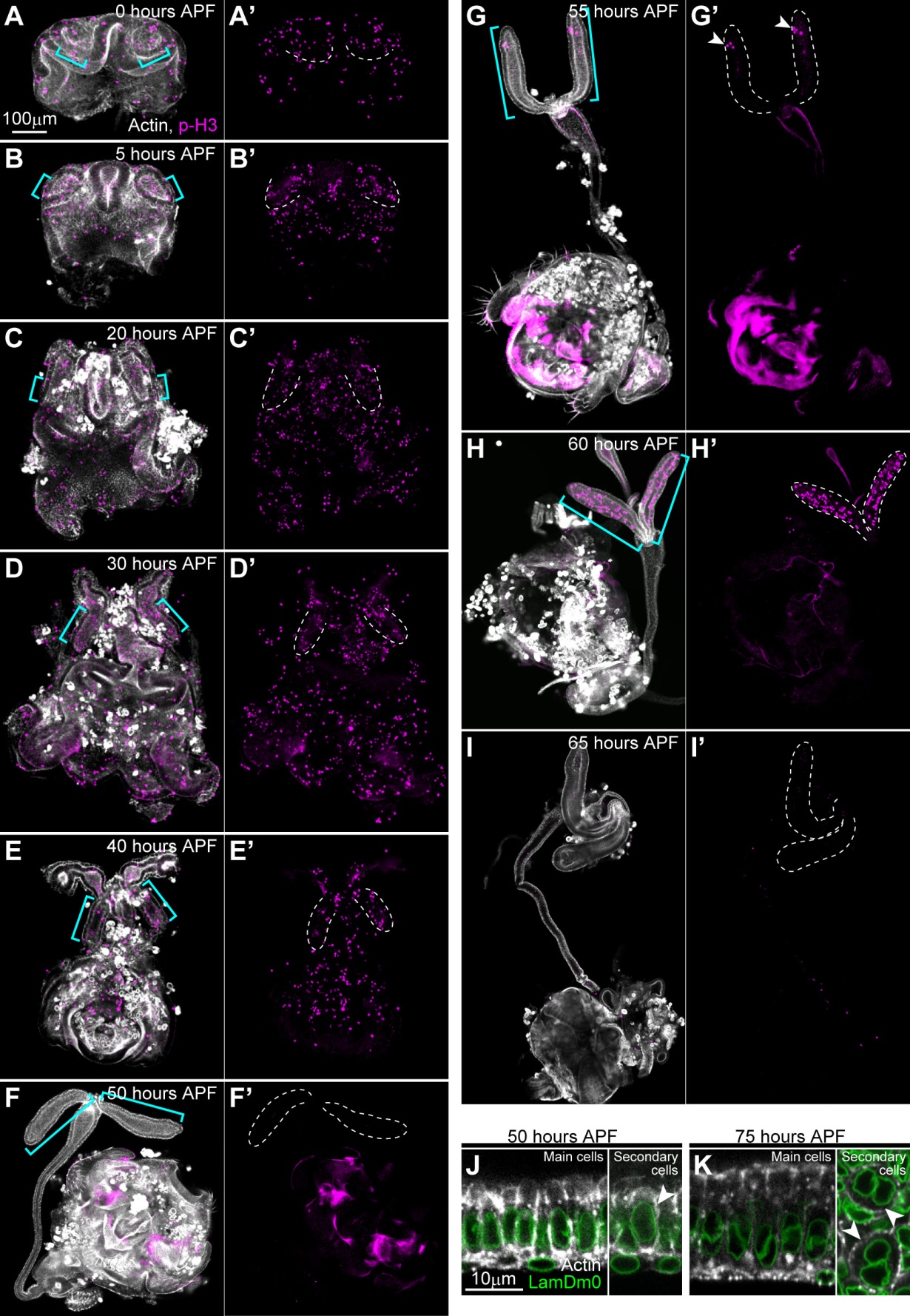
**

**Figure S1** – **Cells in M phase during pupal development of accessory glands**

(A-I and A’-I’) Male reproductive systems labeled with Phalloidin (white) and anti-P-H3 antibody (magenta) at indicated time points. Magenta channels in (A-I) are shown in (A’-I’). Cyan brackets in (A-I) indicate accessory gland primordia. White dashed lines in (A’-H’) indicate outlines of accessory gland primordia. Scale bar in (A), 100 μm, applicable to (A-I and A’-I’). (J and K) Main cells (left) and secondary cells (right, arrowheads) labeled with Phalloidin (white) and anti-LamDm0 antibody for nuclear membrane (green) at 50 (J) and 75 hours APF (K). Cross-sectional views (left and right images in (J) and left image in (K)) and *en face* view (right image in (K)). Scale bar in (J), 10 μm, is applicable to (J and K).

**
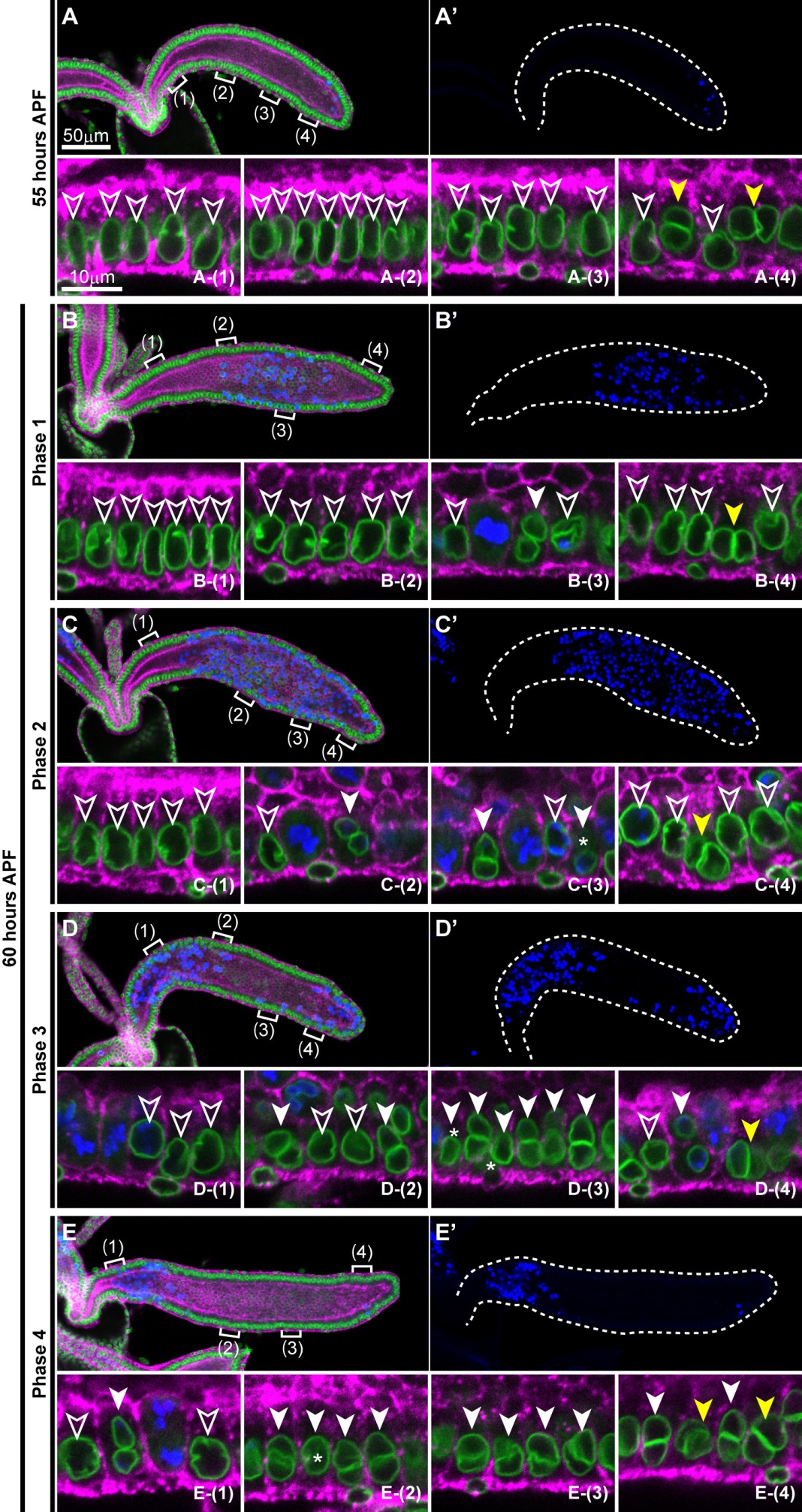
**

**Figure S2** – **Mitotic wave for binucleation of secondary cells and main cells in accessory gland**

(A-E and A’-E’) Male accessory glands labeled with Phalloidin (magenta), anti-LaminDm0 antibody (green) and anti-P-H3 antibody (blue) at 55 h APF (A) and 60 h APF (B-E). Blue channels in (A-E) are shown in (A’-E’). Scale bar in (A), 50 μm, is applicable to (A-E and A’-E’). Magnified images of four regions of the accessory gland lobes ((1): most proximal region, (2): proximal-middle region, (3): distal-middle region and (4): most distal region) are shown at the bottom of (A-E). Binucleate secondary cells (yellow arrowheads), mononucleate main cells (open white arrowheads) and binucleate main cells (closed white arrowheads) are shown. Some of the binucleate cells (marked by asterisks) did not show two nuclei in the displayed confocal section but did in another section. Secondary cells after binucleation can be distinguished by the positioning of two nuclei and cell shape. Scale bar in (A-(1)), 10 μm, is applicable to (A-(1)–(4), B-(1)–(4), C-(1)–(4), D-(1)–(4) and E-(1)–(4)).

(B-E and B-E’) Four developmental stages (phase 1 (A), phase 2 (B), phase 3 (D) and phase 4 (E)) of the male accessory glands based on the distribution of mitotic cells were all collected from the pupae at 60 h APF. The chronological sequence of the four developmental stages can be identified by propagation of binucleation.

**Supporting discussion:**

M-phase entry of the secondary cells for binucleation: (A and A’) During binucleation of the secondary cells at 55 h APF (A-(4)) not all of the main cells have been binucleated yet (A-(1)–(4)). These results suggest that the binucleation of the secondary cells is followed by that of the main cells.

M-phase entry of the main cells for binucleation: When looking at different individuals, the difference in developmental stages in the narrow time window around 60 h APF cannot be detected correctly because the developmental speed varies between the individual pupae. We therefore evaluated accessory glands collected at 60 h APF and identified the following four phases.

(B and B’) Phase 1: In the case in which binucleation was restricted to around the region (3), the cells in other regions did not begin binucleation (B-(1), B-(2), B-(4)). (C and C’) Phase 2: In the case that binucleation was distributed around the regions (2), (3), and (4), only the cells in region (1) did not begin binucleation (C-(1)). (D and D’) Phase 3: In the case that binucleation was distributed around the regions (1), (2), and (4), the cells in region (3) have already finished binucleation (D-(3)). (E and E’) Phase 4: In case that binucleation was observed only around region (1), the cells in the other regions have already finished binucleation (B-(2)-(4)). In all of these cases, binucleation of the secondary cells has completely finished (B-(4)–E-(4)).The results indicate that a mitotic wave for binucleation of the main cells arises at region (3), propagates to more distal and proximal regions, and subsequently split in two. The binucleation in region (1) is initiated at the latest stage.


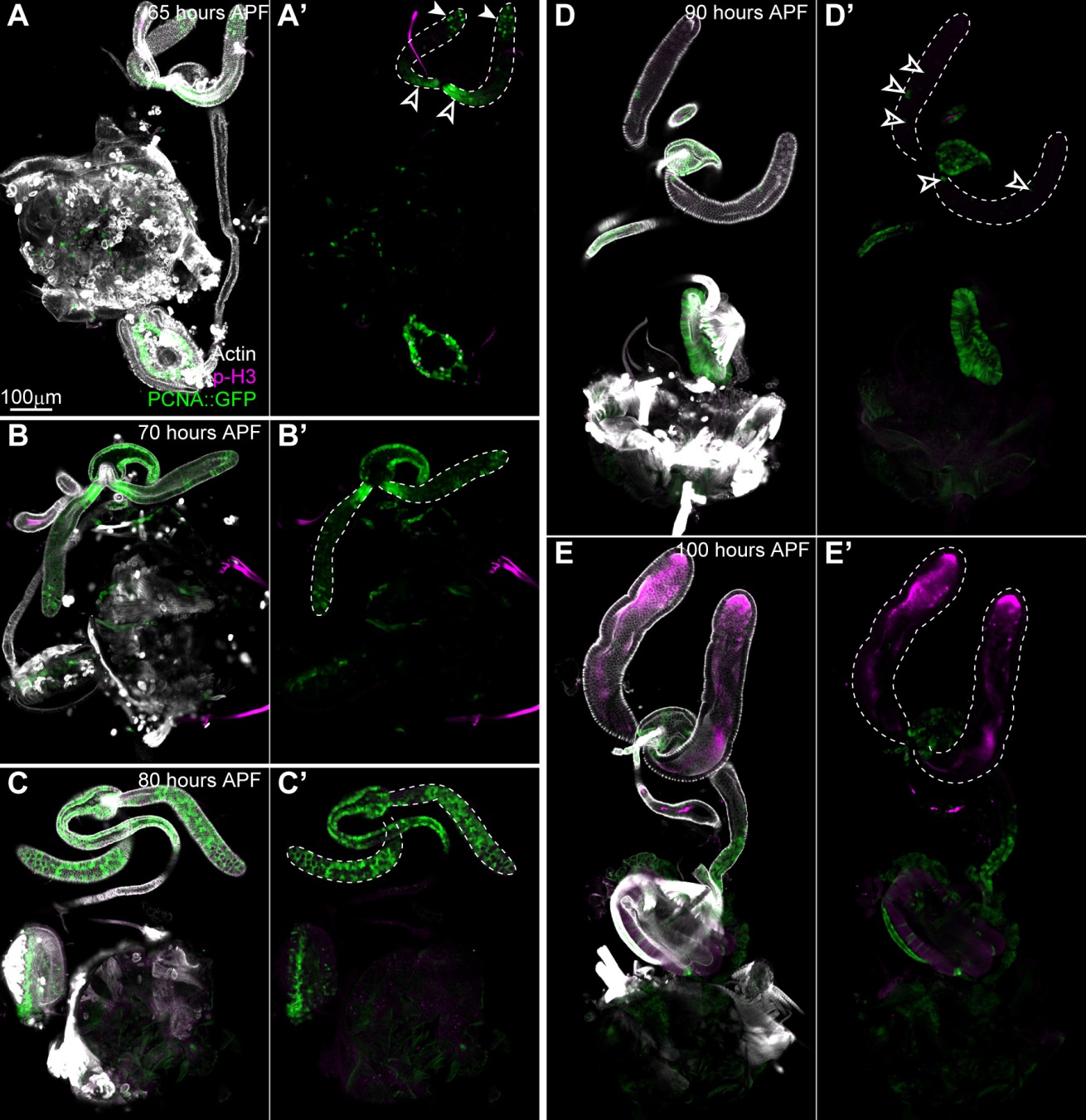


**Figure S3** – **Cells in S and M phases during late pupal development of accessory glands**

(A-E and A’-E’) Male reproductive systems labeled with Phalloidin (white), anti-P-H3 antibody (magenta), and PCNA-GFP (green) at indicated time points. Magenta and green channels in (A-E) are shown in (A’-E’). White dashed lines in (A’-H’) indicate outlines of accessory gland primordia. Highly false-positive staining with anti-P-H3 antibody was observed at the cavity of accessory glands (magenta in (E and E’)). Closed and open arrowheads in (A’ and D’) indicate PCNA-positive secondary and main cells, respectively. Scale bar in (A), 100 μm, is applicable to (A-E and A’-E’).

**
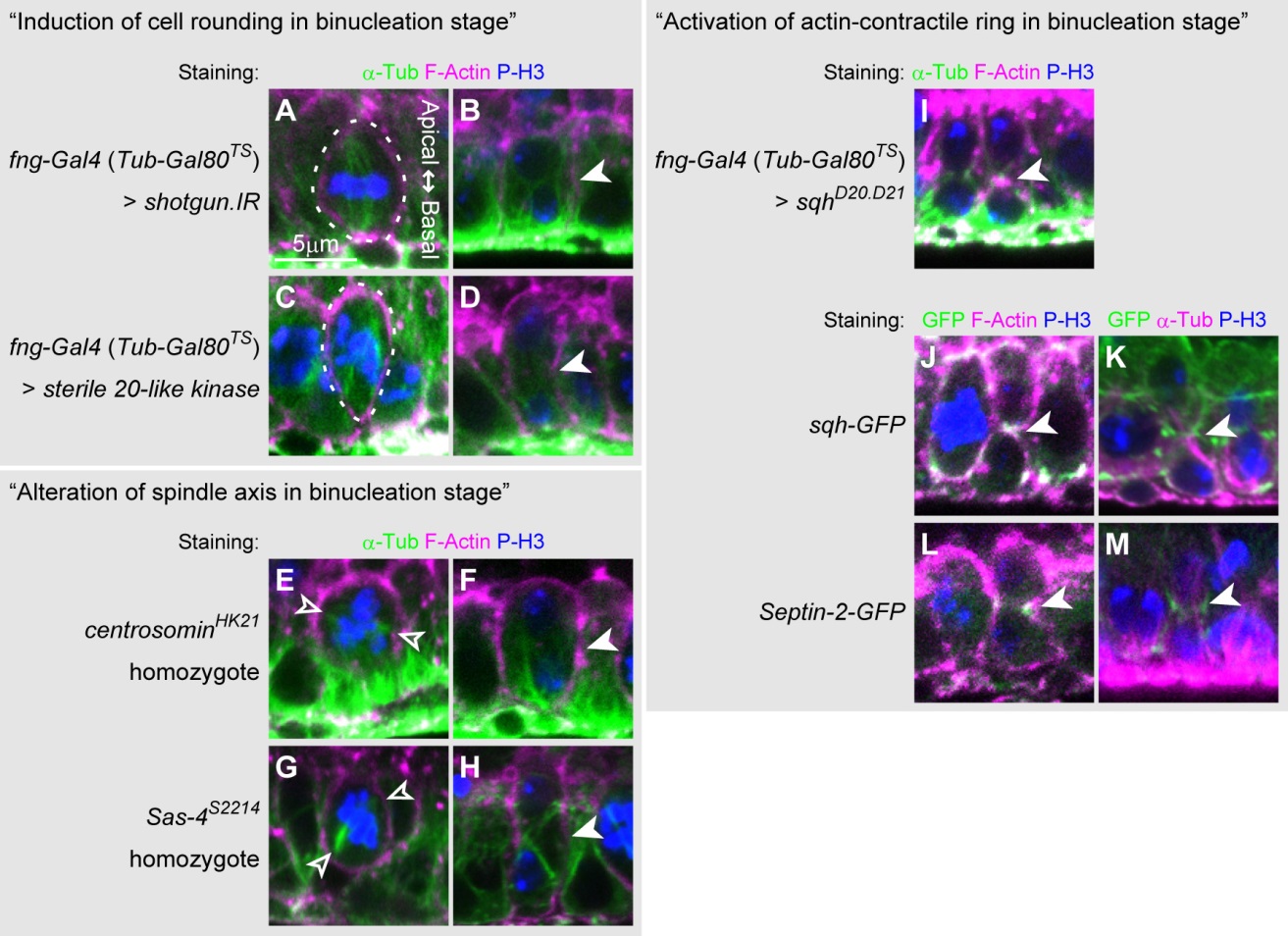
**

**Figure S4** – **Genetic manipulation of cell morphologies in binucleation stage**

(A-M) Binucleation-stage main cells with indicated genotypes. Three types of genetic manipulation ((A-D) induction of cell rounding, (E-H) alteration of the spindle axis, and (I-M) activation of the contractile ring) are shown. Dashed lines indicate outlines of the cells. White arrowheads indicate the equatorial planes. Open arrowheads indicate the spindle poles. Scale bar in (A), 5 μm, is applicable to (A-L). Cells in (A-I) are labeled with Phalloidin (magenta), anti-αTubulin antibody (green) and anti-P-H3 antibody (blue). Cells in (J and L) are labeled with Phalloidin (magenta), GFP (green), and anti-P-H3 antibody (blue). Cells in (K and M) are labeled with anti-αTubulin antibody (magenta), GFP (green), and anti-P-H3 antibody (blue). Cells in (A, C, E and G) are in metaphase and cells in (B, D, F and H-M) are telophase.

**Supporting discussion:**

Knockdown for *shotgun* (A and B) and overexpression of *sterile 20-like kinase* (C and D) induced cell rounding (dashed lines in (A and C)) but did not induce central spindle assembly and contractile ring formation (arrowheads in (B and D)) (also indicated in Table 1). *centrosomin* homozygotes (E and F) and *Sas-4* homozygotes (G and H) showed a defect in vertical spindle orientation (open arrowheads in (E and G)) but did not show central spindle assembly and contractile ring formation (arrowheads in (F and H)) (also indicated in Table 1). These results suggest that the cell rounding or spindle orientation is not involved in the skipping of cytokinesis. Overexpression of the activated form of *sqh* induced formation of the contractile ring and partially assembled central spindle (arrowhead in (I)) (also indicated in Table 1). The *sqh-GFP* transgene (J and K) and *Septin-2-GFP* transgene (L and M) induced formation of the contractile ring, at which *sqh-GFP* and *Septin-2-GFP* were localized, respectively (arrowheads in (J and L)), and the partially assembled central spindle (arrowheads in (K and M)) (Table 1). These results suggest that insufficient activation of the contractile ring formation is involved in the skipping of cytokinesis.


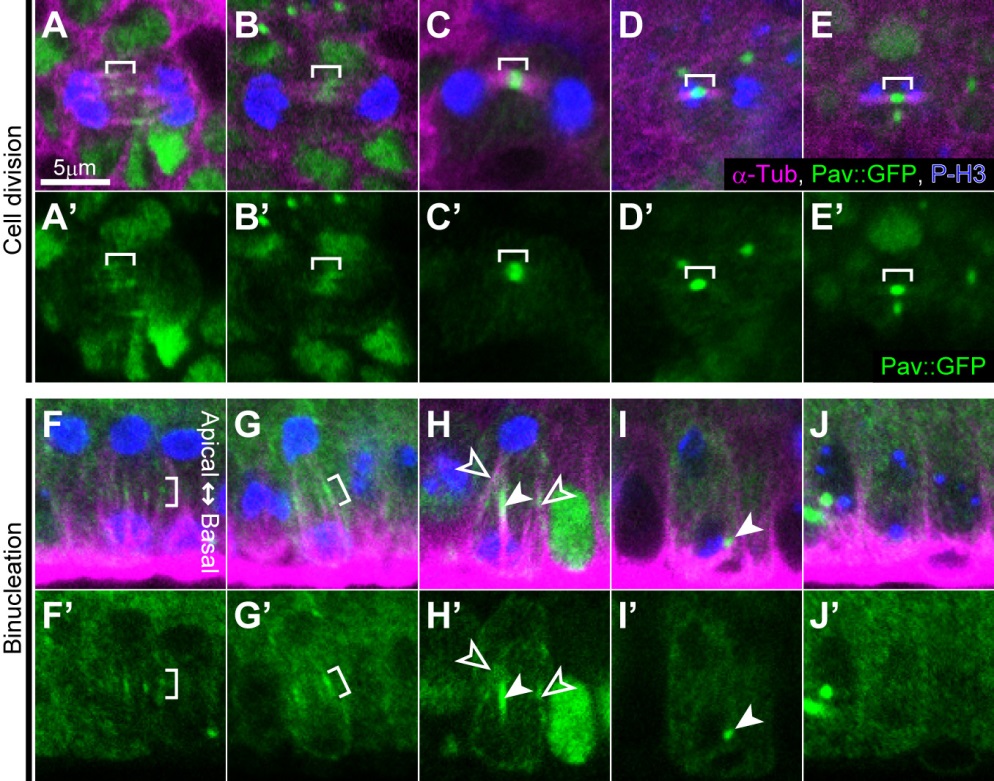


**Figure S5** – **Developing central spindle of binucleating cell is degraded from anaphase to telophase**

*En face* views of the main cells during early pupal cell division stage (A-E and A’-E’) and cross-sectional views of the main cells during the mid-pupal binucleation stage (F-J and F’-J’) with expression of the microtubule plus-end marker Pav::GFP. Cells in anaphase (A-C, A’-C’, F-H, and F’-H’) and telophase (D, E, D’, E’, I, J, I’, and J’) are arrayed from left to right according to the M phase progression. Cells are labeled with anti-α-Tub antibody (magenta), Pav::GFP fluorescence with anti-GFP antibody (green), and anti-P-H3 antibody (blue). Green channels of (A-J) are shown in (A’-J’). Scale bar in (A), 5 μm, is applicable to (A-J and A’-J’).

**Supporting discussion:**

(A, A’) In the process of cell division, during chromosomal segregation at anaphase, Pav::GFP begins to accumulate on microtubules around cell equator (brackets in (A and A’)). (B, C, B’, and C’) At late anaphase, localization of Pav::GFP on microtubule is restricted first to the cell equator on microtubules (brackets in (B and B’)) and subsequently to the midzone (brackets in (C and C’)). This indicates that the plus-ends of microtubules reach the cell equator and the microtubules are bundled to organize the central spindle. (D, E, D’, and E’) This central spindle is maintained during telophase with localization of Pav::GFP at the midzone.

(F, F’) Accumulation of Pav::GFP on microtubules around cell equator during chromosomal segregation is also observed in the process of binucleation (brackets in (F and F’)), suggesting that the assembly of microtubules in middle anaphase is normally initiated. (G, G’, H, and H’) At late anaphase in binucleation, however, Pav::GFP was not sufficiently accumulated at cell equator on the midzone microtubule (brackets in (G and G’)) but was broadly distributed on microtubules, suggesting that the microtubules organizing the central spindle are destabilized. Subsequently, most microtubule bundles are degraded with a broad accumulation of Pav::GFP (open arrowheads in (H and H’)) although some microtubules bundles with strong accumulation of Pav::GFP are more assembled on cell equator (white arrowheads in (H and H’)). However, we never observed the microtubule bundles organized to a single large bundle as occurs in the standard cell division (C and C’). These results imply that in the process of binucleation the microtubule bundles of the central spindle precursor are destabilized and the assembly of the central spindle is impaired. (I, I’, J, and J’) At telophase in binucleation, the central spindle is almost totally degraded although Pav::GFP was sometimes accumulated at the tip of the degraded microtubule bundle (white arrowheads in (I and I’)). Consequently, the microtubule bundles completely disappeared and accumulation of Pav::GFP on microtubules was lost (J and J’). These results suggest that in the process of binucleation the central spindle precursor is degraded in telophase.


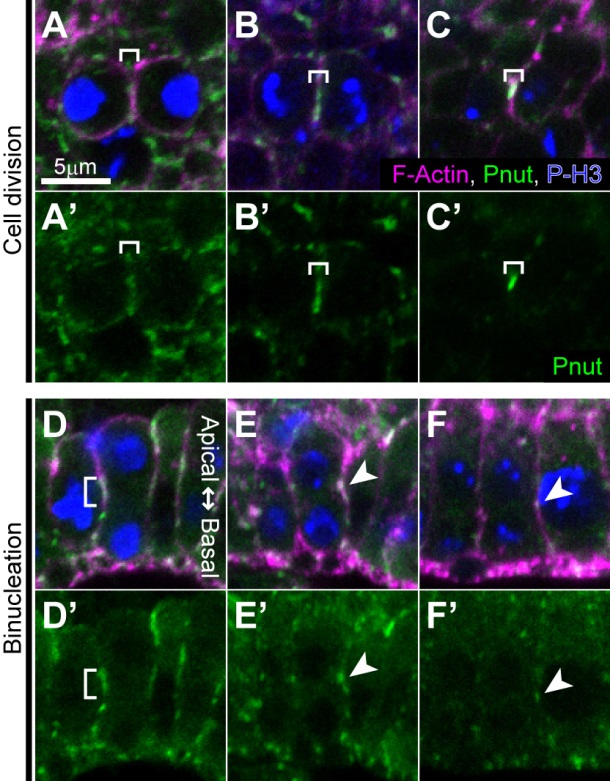


**Figure S6** – **Developing contractile ring of binucleating cell is degraded during telophase**

*En face* views of the main cells during early pupal cell division stage (A-C and A’-C’) and cross-sectional views of the main cells during the mid-pupal binucleation stage (D-F and D’-F’). Cells in anaphase (A, A’, D, and D’) and telophase (B, B’, C, C’, E, E’, F, and F’) are arrayed from left to right according to the M phase progression. Cells are labeled with phalloidin (magenta), anti-Pnut antibody (green), and anti-P-H3 antibody (blue). Green channels of (A-F) are shown in (A’-F’). Scale bar in (A), 5 μm, is applicable to (A-F and A’-F’).

**Supporting discussion:**

(A, A’) In the process of cell division, at anaphase, Pnut begins to accumulate on cleavage furrow (brackets in (A and A’)). (B, C, B’, and C’) At telophase, accumulation of Pnut on cleavage furrow increased (brackets in (B, B’, C, and C’)).

(D, D’) Accumulation of Pav::GFP on microtubules on cleavage furrow is also observed at anaphase in the process of binucleation (brackets in (D and D’)), suggesting that the formation of contractile ring is normally initiated. (E, E’, F, and F’) During telophase in binucleation, however, accumulation of Punt on cleavage furrow was diffused although partial localization of Punt around cell equator often remained (arrowheads in (E, E’, F, and F’)). These results imply that, in the process of binucleation, the contractile ring is regressed during telophase.


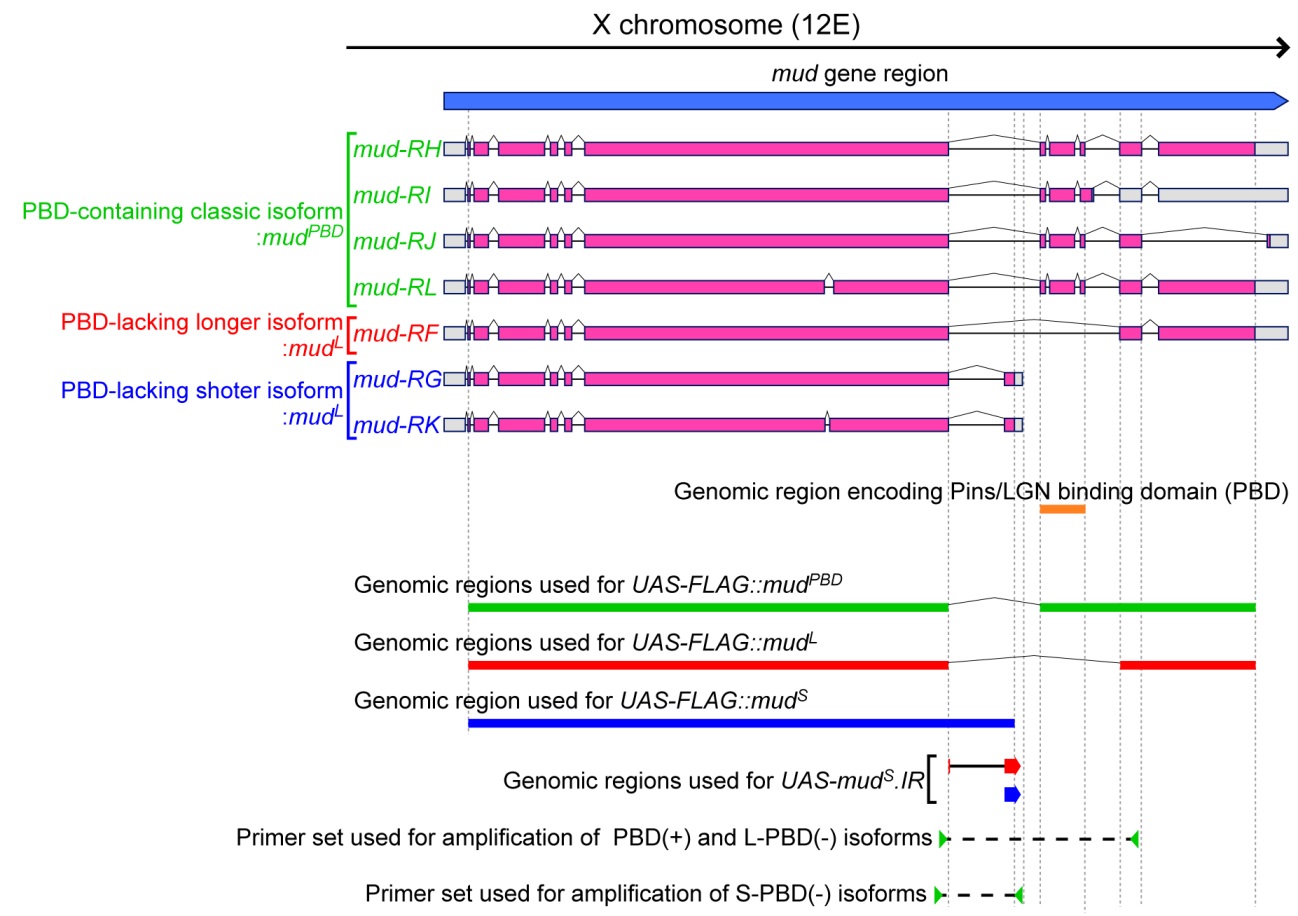


**Figure S7** – **Genomic regions for construction of *UAS-FLAG::mud^PBD^*, *UAS-FLAG::mud^L^*, *UAS-FLAG::mud^S^* and *UAS-mud^S^.IR***

In the upper half, schematic diagram of the *mud* transcriptional unit and splicing variants “*mud-RXs”* are shown. PBD-containing classic isoform (*mud^PBD^*) (RH, RI, RJ and RL), PBD-lacking longer isoform (*mud^L^*) (RF), PBD-lacking shorter isoform (*mud^S^*) (RG and RK) Have benn known. In the lower half, genomic fragment regions used for construction of *UAS-FLAG::mud^PBD^* (green lines) (Figure S8A), *UAS-FLAG::mud^L^* (red lines) (Figure S8A) and *UAS-FLAG::mud^S^* (blue line) (Figure S8A) are shown. Two genomic fragment regions indicated by blue and red small arrows were used for construction of *UAS-mud^S^.IR* d. Primer sets (green arrowheads) used for amplification of *mud^PBD^*,*mud^L^* and *mud^S^* in semi-quantitative RT-PCR (Figure S8C) are also shown.

**
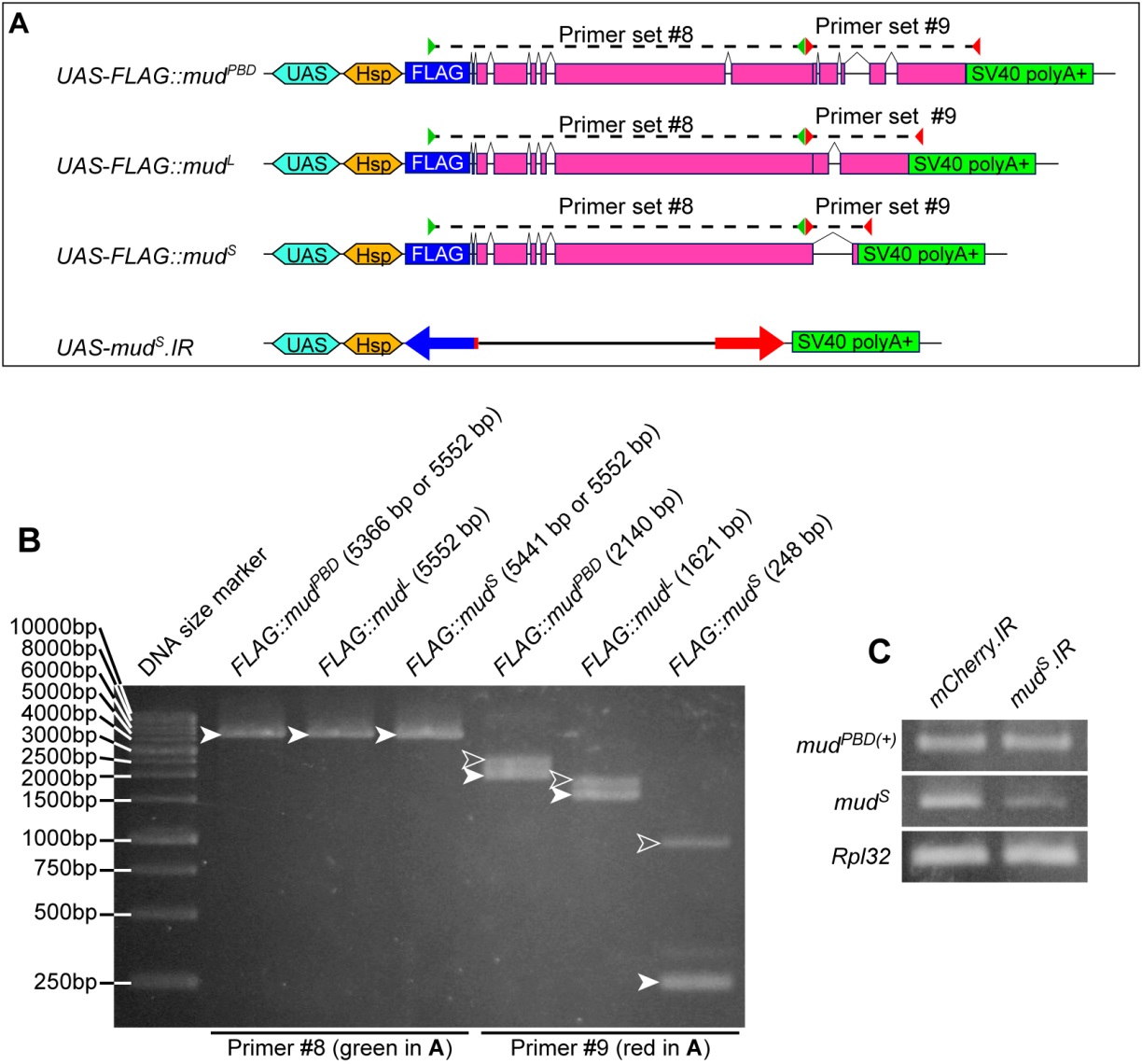
**

**Figure S8** – **Construction and verification of *UAS-FLAG::mud^PBD^*, *UAS-FLAG::mud^L^*, *UAS-FLAG::mud^S^*, and *UAS-mud^S^.IR***

(A) Schematic diagrams showing constructs of *UAS-FLAG::mud^PBD^*, *UAS-FLAG::mud^L^*, *UAS-FLAG::mud^S^*, and *UAS-mud^S^.IR*. Two primer sets (green and red arrowheads) used for amplification of *FLAG::mud* cDNAs in RT-PCR are shown. In *UAS-mud^S^.IR* the reverse complement sequence of the blue arrow in (Figure S7A) and forward sequence of the red arrow in (Figure S7A) were ligated and subcloned into a *pUAS-attB* vector. An intron between the forward and reverse complement primer sequences is expected to be removed by splicing after transcription. (B) RT-PCR for *FLAG::mud^PBD^*, *FLAG::mud^L^*, and *FLAG::mud^S^* transgenes, the expression of which was driven by *hs-Gal4* in the wing imaginal discs. Two PCR-primer sets (green and red arrowheads in A) were used to verify the length of each *mud* cDNA isoform. The overexpressed transgene in each assay and the predicted lengths of the PCR amplicons are indicated above the gel, and the primer sets for PCR are indicated below the gel. Closed arrowheads indicate the PCR amplicons derived from the correctly spliced mRNA of each of the transgenes. Open arrowheads indicate PCR amplicons presumably derived from pre-mRNA because intron-containing transgenes were excessively transcribed in this assay. (C) Semi-quantitative RT-PCR for *mud^PBD^*, *mud^L^*, and *mud^S^* in knockdown of *mCherry* (control) or *mud^S^*. According to the classification shown in Figure S7, PCR amplicons for *mud^PBD^* correspond to *mud-RH*, *mud-RJ*, and *mud-RL* (length, 801 bp) but not to *mud^S^*. PCR amplicons for *mud^L^* (length, 282 bp) were not detected under our experimental conditions, suggesting that this isoform is not expressed in the wing imaginal discs. As a result, *mud^S^.IR* specifically reduced the expression of *mud^S^*. *Rpl32* was used as an internal control.
