# Supplementary material for: Isoform-specific functions of Mud/NuMA mediate binucleation of Drosophila male accessory gland cells
Source: BMC Dev Biol. 2014 Dec 20;14:46. doi: 10.1186/s12861-014-0046-5 (PMC4300151; doi:10.1186/s12861-014-0046-5)
Supplement: Additional file 2: — Tables S1 and S2 are included. [file 12861_2014_46_MOESM2_ESM.docx]

**Table S1** – **Frequencies of cytokinesis characters in four types of cells classified on the basis of cell rounding and horizontal spindle in *mud^4^*mutant.**

| cell types in *mud^4^* | | *frequencies | **Cytokinesis characters | |
| --- | --- | --- | --- | --- |
|  |  |  | Central spindle assembly | Contractile ring formation |
| 1 | Cell rounding (−)  Horizontal spindle (−) | 63% | 81% (N= 22) | 86% (N= 22) |
| 2 | Cell rounding (+)  Horizontal spindle (−) | 29% | 90% (N= 10) | 90% (N= 10) |
| 3 | Cell rounding (−)  Horizontal spindle (+) | 0% |  |  |
| 4 | Cell rounding (+)  Horizontal spindle (+) | 9% | 100% (N= 3) | 100% (N= 3) |
|  | Total of all types | 101% | 86% (N= 35) | 89% (N= 35) |

(+): exhibited, (−): not exhibited

*Frequencies of the types of cells are listed in the third column. The total value 101% is due to rounding.

**Frequencies of cytokinesis characters in each type of cells are listed in the fourth (central spindle assembly) and fifth (contractile ring formation) columns. Data were counted in seven accessory glands.

**Supporting discussion:**

During the binucleation stage, *mud* mutants showed cell-rounding and/or horizontal-spindle phenotypes as well as cytokinesis phenotypes such as central spindle assembly and contractile ring formation. These cytokinesis phenotypes were frequently observed even in cells that did not concurrently exhibit cell-rounding and/or horizontal (abnormal)-spindle phenotypes. These results suggest that the abnormal cytokinesis progression in *mud* mutants is not caused by cell rounding or a horizontal spindle.

**Table S2** – **PCR primers**

| # | PCR target | Primers |
| --- | --- | --- |
| 1 | Upstream enhancer of *Acp70A* | 5’-AGATCTCGGCTATCGAAACATAAAAGTCCC-3’ |
|  |  | 5’-TGCGGCCGCGGTCTGAACACTAAATAAAATATGCGTAGC-3’ |
| 2 | *mud^PBD^* or *mud^L^* | 5’-TGCGGCCGCAAAATGGACTACAAGGACGACGATGACAAGGACACGCGCAGCTGGCGCAAGGTCC-3’ |
|  |  | 5’-TGGTACCCTAATTGCGTCCGGGGCACGAATAGG-3’ |
| 3 | Inverse PCR for *mud^PBD^* | 5’-^Phospho^CGTTCCTTGTATACGGCGGA-3’ |
|  |  | 5’-^Phospho^CATCTTGTTTTTCATCTTCTCCAGC-3’ |
| 4 | Inverse PCR for *mud^L^* | 5’-^Phospho^GACGGACCCCACAGTCTGGATGA-3’ |
|  |  | 5’-^Phospho^CATCTTGTTTTTCATCTTCTCCAGC-3’ |
| 5 | *mud^S^* | 5’-TGCGGCCGCAAAATGGACTACAAGGACGACGATGACAAGGACACGCGCAGCTGGCGCAAGGTCC-3’ |
|  |  | 5’-TGGTACCCTAATACACGATAGGACTGCGCGAC-3’ |
| 6 | Genomic fragment with intron  for *mud^S^.IR* | 5’-TGCGGCCGCCAAGATGGTAAGTCGCTGCG-3’ |
|  |  | 5’-ACTCGAGACAAAGTCTACCACGTCATGTG-3’ |
| 7 | Genomic fragment  for *mud^S^.IR* | 5’-TGAATTCCTAATACACGATAGGACTGCGC-3’ |
|  |  | 5’-AGCGGCCGCAAGGGGTCACGCCGTCGGCG-3’ |
| 8 | RT-PCR for  *UAS* targeted *mud* isoforms-N | 5’-CTACAAGGACGACGATGACA-3’ |
|  |  | 5’-GCGCTAGCTTTTGGTATCTC-3’ |
| 9 | RT-PCR for  *UAS* targeted *mud* isoforms-C | 5’-GAAGATAAAGGACCAGCGTC-3’ |
|  |  | 5’-CACCACAGAAGTAAGGTTCC-3’ |
| 10 | RT-PCR for *Rpl32* | 5’-AGATCGTGAAGAAGCGCACCAAG-3’ |
|  |  | 5’-CCGGATTCAAGAAGTTCCTGGTG-3’ |
| 11 | RT-PCR  for *mud^PBD^* & *mud^L^* | 5’-TTGAGATGGAGGGAAAGCTG-3’ |
|  |  | 5’-TGAATCGCTGCGTCAGGAAC-3’ |
| 12 | RT-PCR for *mud^S^* | 5’-AAAAGCTAGCGCTAGACTGC-3’ |
|  |  | 5’-AGTCTACCACGTCATGTGTG-3’ |
